# Sequential soxhlet fractionation of *Cistus ladanifer* L. reveals a polarity–activity gradient: GC-MS profiling, multimechanistic bioactivity, and in silico identification of antifungal and antibacterial lead compounds

**DOI:** 10.3389/fchem.2026.1909751

**Published:** 2026-08-05

**Authors:** Bahia Abdelfattah, Oussama Khibech, Amena Mrabet, Jaber Maataoui, Amr Kchikich, Abdelaaty A. Shahat, Rashed N. Herqash, Joe Miantezila Basilua, Mohamed Bouhrim, Asmae El Cadi, Mohamed Khaddor

**Affiliations:** 1 Chemistry Department, Laboratory of Physical Chemistry of Materials, Natural Substances and Environment (LAMSE), Faculty of Sciences and Techniques of Tangier, Tangier, Morocco; 2 Laboratory of Applied Chemistry and Environment (LCAE), Faculty of Science, University Mohammed Premier, Oujda, Morocco; 3 National Sheep and Goat Association (ANOC), Mers ElKheir, Skhirat-Temara, Morocco; 4 Center for Research on Medicinal, Aromatic, and Poisonous Plants, DSR, King Saud University, Riyadh, Saudi Arabia; 5 Department of Biostatistics and Mathematics, Faculty of Pharmacy, Paris Cité University, Paris, France; 6 Biological Engineering Laboratory, Faculty of Sciences and Techniques, Sultan Moulay Slimane University, Beni Mella, Morocco

**Keywords:** ADMET, antibacterial activity, antifungal activity, antioxidant activity, *Cistus ladanifer*, FTIR, GC-MS, molecular docking

## Abstract

**Introduction:**

AMR and oxidative stress are among the most pressing biomedical challenges, requiring structurally novel multi-target bioactive molecules. *Cistus ladanifer* L. is an ethnobotanical resource whose polarity-resolved phytochemical profile remains largely incomplete.

**Methods:**

*C. ladanifer* leaves (Rmilate forest, Tangier) were fractionated by sequential Soxhlet extraction (n-hexane, chloroform, ethanol, water) and evaluated by phytochemical quantification, FTIR, GC-MS, four antioxidant assays (DPPH, ABTS, FRAP, ORAC), antibacterial well-diffusion/microdilution (*S. aureus, B. subtilis, E. coli, P. aeruginosa*), antifungal assays (*T. rubrum, M. canis, B. cinerea, F. oxysporum*), and ADMET/docking of four marker compounds (EOMCP, DHMAQ, esculin, diosgenin).

**Results:**

A strict polarity-dependent gradient was observed, with the aqueous fraction showing the highest phenolic (140 mg GAE/g DW), flavonoid (148 mg QE/g DW), and tannin (210 mg TAE/g DW) content; strongest antioxidant activity (DPPH IC50 = 0.09 mg/mL; FRAP = 65.25 mg TE/g DW); lowest MIC (0.25 mg/mL, bactericidal, MBC/MIC ≤4); and highest antifungal inhibition (86.0% against *T. rubrum* at 4 mg/mL). Diosgenin showed the strongest docking affinity against CYP51B (5FRB, −12.7 kcal/mol), exceeding the reference ligand.

**Discussion:**

These findings establish a polarity–activity framework supporting *C. ladanifer's* valorization as an antioxidant, antibacterial, and antifungal resource, pending cytocompatibility and *in vivo* evaluation.

## 1 Introduction

Antimicrobial resistance (AMR) and oxidative stress represent two of the most formidable and interconnected challenges confronting contemporary biomedical research. The World Health Organization has classified AMR as a global health emergency, with drug-resistant bacterial pathogens now responsible for an estimated 1.27 million direct deaths and contributing to approximately 4.95 million fatalities annually worldwide (Murray et al., 2022 ; Khare et al., 2021). The rapid and unchecked dissemination of multidrug-resistant (MDR) pathogens—including carbapenem-resistant Enterobacteriaceae, methicillin-resistant *Staphylococcus aureus* (MRSA), and extensively drug-resistant *Pseudomonas aeruginosa*—has rendered an expanding proportion of clinical infections refractory to the existing antibiotic armamentarium, threatening to reverse decades of progress in infectious disease management. Concurrently, the dysregulation of cellular redox homeostasis and the consequent accumulation of reactive oxygen species (ROS) have been mechanistically implicated in the initiation and progression of a broad spectrum of non-communicable diseases, including cancer, type 2 diabetes, cardiovascular disease, neurodegenerative disorders, and accelerated biological aging (Newman and Cragg, 2020; Atanasov et al., 2021). Both challenges share a common pharmacological imperative: the urgent identification of novel bioactive molecules with multi-target efficacy, chemical scaffold diversity that minimizes cross-resistance, and safety profiles compatible with long-term therapeutic use.

Medicinal plants constitute one of the most structurally and mechanistically rich repositories of pharmacologically active natural products, and their systematic phytochemical investigation continues to yield lead compounds of clinical and nutraceutical relevance that have not yet been replicated by combinatorial synthetic chemistry (Newman and Cragg, 2020). Of the therapeutically approved small molecules introduced between 1981 and 2019, more than one-third derive directly or structurally from natural product scaffolds, with plant-derived compounds accounting for a disproportionate share of anti-infective and antioxidant agents (Newman and Cragg, 2020; Atanasov et al., 2021). The Mediterranean Basin, designated one of the world’s 36 recognized biodiversity hotspots, harbors a particularly exceptional phytochemical diversity attributable to its complex paleoclimatic history, edaphic heterogeneity, and the evolutionary pressure exerted by long-standing co-evolutionary interactions between plants and herbivores, pathogens, and environmental stressors (Nieto Feliner et al., 2023; Kessler and Kalske, 2018). The aromatic and resinous shrub flora endemic to this region, shaped by millennia of adaptation to high ultraviolet irradiance, seasonal drought, and microbial challenge, has evolved a remarkable capacity to biosynthesize structurally complex polyphenols, terpenoids, and phenylpropanoids with potent antioxidant, antimicrobial, and anti-inflammatory activities—chemical profiles that translate directly into pharmacological relevance for the therapeutic areas most affected by the AMR and oxidative stress crises (Laoué et al., 2022).

*Cistus ladanifer L.* (Cistaceae), commonly known as gum rockrose or labdanum cistus, is a resinous, aromatic shrub endemic to the western Mediterranean region and widely distributed across the Iberian Peninsula, Morocco, Algeria, and adjacent Atlantic-facing territories, including habitats in the Rmilate forest near Tangier, Morocco (Abdelfattah et al., 2025; Abdelfattah et al., 2024). The species occupies a dominant ecological role in garrigue and matorral phytocommunities, particularly on acidic, nutrient-poor soils following fire disturbance, and is distinguished by the copious production of a viscous oleoresin—labdanum—exuded from glandular trichomes on its leaves and stems (Papaefthimiou et al., 2014; Gomes et al., 2011; Adadi et al., 2021). Ethnobotanically, C. ladanifer occupies a prominent position in North African and Ibero-Moroccan folk medicine, where it is traditionally employed as an aqueous infusion or decoction for the treatment of skin infections, wound healing, gastrointestinal disorders, respiratory conditions, and inflammatory ailments—therapeutic applications that collectively point toward the presence of potent antimicrobial and antioxidant secondary metabolites (Bouothmany et al., 2022; El Karkouri et al., 2021; Köse et al., 2017). The plant’s phytochemical signature is dominated by a diverse array of polyphenolic compounds, including hydrolyzable tannins (notably punicalagin, casuariin, and punicalin), ellagic acid and its glycoside derivatives, gallic acid, quercetin-3-O-glucoside, kaempferol-3-O-rutinoside, hydroxycinnamic acid esters, labdane-type diterpenes (labdanolic acid, cistadiol), and an extensive suite of volatiles comprising monoterpenes (camphene, α-pinene, β-pinene) and sesquiterpenes (Papaefthimiou et al., 2014; Bakrim et al., 2021; Abdelfattah et al., 2024; Barrajón-Catalán et al., 2011). This exceptional chemical richness underpins the growing scientific interest in C. ladanifer as a multi-target pharmacological resource.

Despite the established ethnomedicinal reputation of Cistus ladanifer and a growing body of preliminary phytochemical literature, the systematic translation of its bioactive potential into reproducible, polarity-resolved pharmacological data remains conspicuously incomplete. Existing studies have predominantly focused on volatile essential oil fractions or crude hydroalcoholic extracts, providing only fragmented insights into the differential bioactivity contributed by chemically distinct phytochemical classes (Zazouli and Mannani, 2026; Alves-Ferreira et al., 2022). Critically, no study to date has subjected C. ladanifer leaf material to sequential Soxhlet fractionation using a continuous polarity gradient from strictly apolar to aqueous solvents—a preparative approach that, by operating under thermodynamic equilibrium conditions at the boiling point of each solvent, ensures exhaustive and reproducible chemical partitioning according to compound polarity, hydrogen-bonding capacity, and lipophilicity. Furthermore, the systematic integration of quantitative phytochemical profiling (total phenolics, flavonoids, and tannins), FTIR spectroscopic characterization, GC-MS compositional mapping, and multimechanistic bioactivity evaluation (antioxidant, antibacterial, and antifungal) across all fractions of a single Moroccan accession has not previously been reported. This absence of polarity-resolved data constitutes a fundamental obstacle to rationalizing the ethnobotanical activity of this plant, to identifying which chemical domains are responsible for specific biological effects, and to establishing a scientifically sound basis for the selective valorization of its extracts in pharmaceutical, nutraceutical, and cosmetic applications.

Sequential Soxhlet extraction using solvents of progressively increasing polarity—n-hexane, chloroform, ethanol, and water—represents one of the most rigorous and reproducible methodologies available for the systematic chemical fractionation of complex plant matrices (Cao et al., 2025; Sun et al., 2025). This approach leverages the thermodynamic principle of ‘like dissolves like’ to achieve a near-complete segregation of plant secondary metabolites into chemically coherent fractions: strictly apolar solvents selectively recover cuticular waxes, alkanes, fixed oils, and lipophilic terpenoids; solvents of intermediate polarity enrich flavonoid aglycones, coumarins, and semi-polar phenylpropanoids; polar protic solvents solubilize flavonoid glycosides, phenolic acids, and oligomeric tannins; and hot water extracts hydrophilic metabolites including polysaccharides, uronic acids, and hydrolyzed tannin precursors (Rodríguez De Luna et al., 2020; Sun et al., 2025). When coupled with comprehensive phytochemical quantification and multimechanistic bioactivity assays, this polarity-stratified framework enables the direct mapping of structure-activity relationships across chemically defined extract domains, thereby transforming crude ethnobotanical observations into mechanistically grounded pharmacological evidence. The pairing of this fractionation strategy with *in silico* ADMET analysis and molecular docking of selected marker compounds further extends the translational relevance of the experimental findings by providing an early-stage assessment of the drug-likeness and target-binding potential of the most pharmacologically significant constituents identified by GC-MS.

The present study was therefore designed to provide a comprehensive, polarity-resolved phytochemical and bioactivity profile of Cistus ladanifer L. leaves collected from the Rmilate forest, Tangier, Morocco. Specifically, the objectives were: (i) to prepare four sequential Soxhlet fractions (hexane, chloroform, ethanol, and aqueous) and determine their extraction yields; (ii) to quantify total phenolic, flavonoid, and tannin contents across all fractions; (iii) to characterize the molecular functional group composition of each fraction by FTIR spectroscopy; (iv) to identify and comparatively profile the volatile and semi-volatile phytochemical constituents of each fraction by GC-MS; (v) to evaluate the antioxidant activity of each fraction through four complementary mechanistic assays (DPPH, ABTS, FRAP, and ORAC); (vi) to assess the antibacterial activity against a clinically representative panel of Gram-positive and Gram-negative bacteria, including determination of minimum inhibitory and minimum bactericidal concentrations; (vii) to evaluate antifungal activity against both dermatophytic and phytopathogenic fungal strains; and (viii) to perform *in silico* ADMET profiling and molecular docking of selected marker compounds against relevant antibacterial and antifungal target proteins. Collectively, this integrated experimental and computational approach aims to establish a rigorous scientific basis for the rational valorization of C. ladanifer as a source of biologically active phytochemicals for pharmaceutical and nutraceutical development.

## 2 Materials and methods

### 2.1 Plant material

Cistus ladanifer L. plants were collected in March 2025 from the Rmilate forest, Tangier, Morocco (35.7876°N, 5.8653°W), situated in the northern region of the country. Healthy, mature specimens were selected at the flowering (anthesis) stage on the basis of visual morphological criteria. The number of individual shrubs sampled was not recorded, and the harvested leaves were pooled without counting. Botanical identification was performed by Prof. Mohamed El Kadiri, and a voucher specimen (No. LAMSE-CL-25–001) was subsequently deposited at the university herbarium for future reference. Fresh leaves were manually separated from the stems and subjected to shade-drying at ambient temperature for 15 days until complete desiccation was attained. The dried plant material was then stored in sealed paper bags under controlled dry conditions, protected from light and moisture, until further use.

### 2.2 Preparation of plant extracts by soxhlet apparatus

The dried plant material was ground into a fine, homogeneous powder and passed through a 1 mm mesh sieve. A 60 g portion of the dried powder was subjected to sequential Soxhlet extraction using 250 mL of each solvent, arranged in order of increasing polarity: *n*-hexane, chloroform, ethanol, and distilled water. This procedure yielded four distinct fractions, designated as hexane (Hex), chloroform (Chl), ethanol (EtOH), and aqueous (AQ). Each obtained extract was filtered through Whatman No. 1 filter paper and subsequently evaporated to dryness under reduced pressure at ≤40 °C using a rotary evaporator. The resulting dry residues were weighed to determine the extraction yield (%), calculated as the ratio of dry extract mass to the initial powder mass (w/w). All extracts were transferred into sealed amber glass vials and stored at 4 °C in the dark until further analysis. The extraction yields, expressed as a percentage of the initial dry powder mass (w/w), were 7.60% (hexane), 5.02% (chloroform), 13.43% (ethanol), and 19.27% (aqueous). Extraction with each solvent was continued for approximately 6–8 h, until the siphoning eluent ran colourless, ensuring exhaustive extraction of each fraction.

### 2.3 Phytochemical evaluation

Polyphenols, flavonoids, and tannins represent major classes of bioactive secondary metabolites widely distributed in medicinal plants, and are well recognized for their potent antioxidant, anti-inflammatory, and antiproliferative properties. Their accurate quantification is considered fundamental to evaluating the therapeutic potential of plant-derived extracts and to informing evidence-based phytopharmaceutical formulation strategies.

#### 2.3.1 Total polyphenol content (TPC)

Total phenolic content (TPC) was determined using the Folin–Ciocalteu colorimetric method as previously described by Abdelfattah et al. (2024), with minor modifications. Briefly, 100 µL of each Soxhlet extract (1 mg/mL) was combined with 400 µL of Folin–Ciocalteu reagent (diluted in distilled water), followed by the addition of 1 mL of 7% (w/v) sodium carbonate (Na_2_CO_3_) solution. The reaction mixture was then brought to a final volume of 1.6 mL with distilled water and allowed to incubate for 30 min at room temperature under dark conditions. Absorbance was measured at 765 nm using a UV–Vis spectrophotometer. A calibration curve was established using gallic acid as the reference standard (y = 17.5x + 0.1113; R^2^ = 0.9985), and results were expressed as milligrams of gallic acid equivalents per gram of dry weight (mg GAE/g DW).

#### 2.3.2 Total flavonoid content (TFC)

Total flavonoid content (TFC) was determined using the aluminum chloride (AlCl_3_) colorimetric method as previously described by Mrabet et al. (2024a). Briefly, 200 µL of each Soxhlet extract was added to 1800 µL of a reagent solution prepared by combining 1 mL of potassium acetate (1 M), 1 mL of aluminum chloride (10%, w/v), 10 mL of methanol (50%, v/v), and 24 mL of distilled water. The resulting mixture was allowed to incubate for 30 min at room temperature under dark conditions, after which absorbance was measured at 415 nm using a UV–Vis spectrophotometer. Quercetin was used as the reference standard for calibration curve construction (y = 5.9566x + 0.1353; R^2^ = 0.9999), and results were expressed as milligrams of quercetin equivalents per gram of dry weight (mg QE/g DW).

#### 2.3.3 Total tannin content (TTC)

Total tannin content (TTC) was determined using the Folin–Ciocalteu colorimetric method as previously described by Mrabet et al. (2024b), with minor modifications. Briefly, 100 µL of each Soxhlet extract was combined with 0.5 mL of Folin–Ciocalteu phenolic reagent and 1 mL of 30% (w/v) sodium carbonate (Na_2_CO_3_) solution. Following thorough vortex mixing, the reaction mixture was allowed to incubate at room temperature under dark conditions for 30 min, after which absorbance was measured at 700 nm using a UV–Vis spectrophotometer. A calibration curve was established using tannic acid as the reference standard (y = 3.6129x + 0.1806; R^2^ = 0.9933), and results were expressed as milligrams of tannic acid equivalents per gram of dry weight (mg TAE/g DW).

### 2.4 Chemical characterization

#### 2.4.1 FTIR spectroscopy

Fourier-transform infrared (FTIR) spectroscopy was employed to characterize the chemical composition and molecular structure of both the raw plant material and its corresponding Soxhlet-derived extracts. Samples were prepared as potassium bromide (KBr) pellets by homogeneously blending a precisely weighed quantity of each extract with dried KBr, followed by compression under high pressure to produce transparent discs suitable for spectroscopic analysis. Spectra were acquired over a wavenumber range of 400–4,000 cm^-1^ at a spectral resolution of 4 cm^-1^, with each spectrum accumulated over 32 co-added scans to maximize the signal-to-noise ratio and ensure data reproducibility.

#### 2.4.2 GC-MS analysis

Prior to GC-MS analysis, extracts were subjected to appropriate derivatization procedures based on their respective polarity profiles. Polar Soxhlet extracts were derivatized via silylation following the protocols described by Muazu et al. (2022), with minor modifications. Briefly, 5 mg of each extract was dissolved in 200 µL of pyridine, combined with 200 µL of N,O-bis(trimethylsilyl)trifluoroacetamide (BSTFA) containing 1% trimethylchlorosilane (TMCS), and vortexed thoroughly. The mixture was subsequently heated at 70 °C for 2 h and cooled at 4 °C for 15 min. Thereafter, 400 µL of n-hexane was added, and the mixture was centrifuged at 5,000 rpm for 5 min. The resulting supernatant was carefully transferred into GC vials for subsequent analysis. Apolar extracts were transesterified according to the method of Metcalfe et al. (1966), with slight modifications, involving sequential treatment with methanolic sodium hydroxide (NaOH, 0.5 M) and boron trifluoride/methanol (BF_3_/methanol, 10%) under reflux at 65 °C, followed by liquid–liquid extraction with dichloromethane and drying over anhydrous sodium sulfate (Na_2_SO_4_).

GC-MS analysis was performed using a Thermo Scientific TRACE 1300 gas chromatograph coupled to a TSQ 8000 Evo triple quadrupole mass spectrometer, equipped with a TR-5 fused-silica capillary column (30 m × 0.25 mm × 0.25 µm film thickness). High-purity helium was used as the carrier gas at a constant flow rate of 1.5 mL/min. A programmable split/splitless injector was maintained at 250 °C, with a split injection volume of 1.0 µL. The column oven temperature was programmed from 70 °C to 300 °C at a ramp rate of 4 °C/min, with a final isothermal hold of 30 min. Electron ionization (EI) was applied at an ionization energy of 70 eV. Compounds were tentatively identified by matching experimental mass spectra and calculated retention indices against reference entries in the NIST mass spectral library (NIST 2020); in the absence of co-injected authentic standards, all assignments are considered tentative. Representative total ion chromatograms of the four Soxhlet fractions are provided as Supplementary Material (Supplementary Figures S1–S4). Dedicated solvent blank chromatograms were not acquired; to limit the risk of solvent- or column-derived artefacts, annotations were restricted to peaks with high NIST match factors and consistent calculated retention indices, and none of the annotated constituents corresponded to characteristic column-bleed or common laboratory contaminants (e.g., siloxanes or phthalates).

### 2.5 Biological activities

#### 2.5.1 Antioxidant activity

The antioxidant potential of the Soxhlet extracts was evaluated through four established assays—DPPH and ABTS radical scavenging, ferric reducing antioxidant power (FRAP), and oxygen radical absorbance capacity (ORAC) — covering both hydrogen atom transfer and electron transfer mechanisms.

#### 2.5.2 DPPH radical scavenging assay

The free radical scavenging activity of the Soxhlet extracts was evaluated using the 2,2-diphenyl-1-picrylhydrazyl (DPPH) assay as previously described by Kosanic et al. (2014), with minor modifications. Serial dilutions of each extract were prepared in methanol at concentrations ranging from 0.015625 to 1 mg/mL. Aliquots of 250 µL of each dilution were combined with 750 µL of DPPH methanolic solution and allowed to incubate for 30 min at room temperature under dark conditions. Absorbance was measured at 517 nm using a UV–Vis spectrophotometer, and the percentage of radical scavenging inhibition was calculated based on the difference in absorbance between the blank control and the tested extract solution. The half-maximal inhibitory concentration (IC_50_), defined as the extract concentration required to inhibit 50% of DPPH radical activity, was determined by nonlinear regression analysis and used as a quantitative measure of antioxidant potency. Ascorbic acid was included as a positive reference control. All assays were conducted in triplicate, and results are expressed as mean ± standard deviation (SD).

#### 2.5.3 ABTS radical scavenging assay

The ABTS radical scavenging activity of the Soxhlet extracts was assessed using the method of Abdelfattah et al. (2024), with minor modifications. The ABTS radical cation (ABTS•^+^) stock solution was prepared by dissolving 7 mM 2,2′-azino-bis(3-ethylbenzothiazoline-6-sulfonic acid) (ABTS) in distilled water and adding 2.45 mM potassium persulfate, followed by incubation in the dark at room temperature for 12–16 h to allow complete and stable radical formation. Prior to use, the ABTS•^+^ stock solution was diluted with absolute ethanol to achieve an absorbance of 0.70 ± 0.02 at 734 nm. Serial dilutions of each extract were prepared at concentrations ranging from 0.015625 to 1 mg/mL. Thereafter, 75 µL of each extract dilution was combined with 925 µL of the ABTS•^+^ working solution and allowed to incubate for 10 min at room temperature under dark conditions. Absorbance was measured at 734 nm, and the percentage of radical scavenging inhibition was calculated based on the difference in absorbance between the blank control and the tested extract solution. The IC_50_ value was determined by nonlinear regression analysis, and ascorbic acid was included as a positive reference control. All measurements were conducted in triplicate, and results are expressed as mean ± standard deviation (SD).

#### 2.5.4 Ferric reducing antioxidant power (FRAP) assay

The reducing power of the Soxhlet extracts was assessed using the FRAP assay as described by Arroussi et al. (2022). The FRAP reagent was freshly prepared by mixing 10 mM TPTZ (dissolved in 40 mM HCl), 20 mM FeCl_3_, and 300 mM acetate buffer (pH 3.6) in a 10:1:1 ratio, and pre-incubated at 30 °C. Diluted extracts were added to the pre-warmed FRAP reagent in a microplate, and absorbance was measured at 593 nm. A Trolox calibration curve was used, and results were expressed as milligrams of Trolox equivalents per gram of dry weight (mg TE/g DW).

#### 2.5.5 Oxygen radical absorbance capacity (ORAC) assay

The oxygen radical absorbance capacity (ORAC) assay was performed following the method of Escribano et al. (2017). Briefly, 120 µL of fluorescein solution (80 nM in 13.3 mM phosphate-buffered saline, PBS) was added to each well of a microplate containing the extract. Following initial fluorescence recording, 2,2′-azobis (2-amidinopropane) dihydrochloride (AAPH) was introduced as a peroxyl radical generator, and fluorescence decay was monitored at 2 min intervals over 120 min. ORAC values were derived from the area under the fluorescence decay curve using Gen5 2.0 software (BioTek Instruments, Winooski, VT, USA) as described by Mendes et al. (2016), and expressed as milligrams of Trolox equivalents per gram of dry weight (mg TE/g DW).

#### 2.5.6 Antibacterial activity

The antibacterial activity of *Cistus ladanifer* Soxhlet extracts was evaluated using the agar well diffusion method following a standardized procedure with minor modifications (Gonzalez-Pastor et al., 2023). Four reference bacterial strains were tested: two Gram-positive (*Staphylococcus aureus* ATCC 25923 and *Bacillus subtilis* ATCC 6633) and two Gram-negative (*Escherichia coli* ATCC 25922 and *Pseudomonas aeruginosa* ATCC 27853). Bacterial suspensions were prepared in sterile saline and adjusted to the 0.5 McFarland standard (≤10^8^ CFU/mL). Mueller–Hinton agar plates were uniformly inoculated by surface swabbing, after which 6 mm wells were aseptically punched and filled with 25 µL of each extract. Extracts were dissolved in dimethyl sulfoxide (DMSO) to prepare stock solutions and diluted in the assay medium prior to testing. Gentamicin (10 μg/mL) served as the positive control, while DMSO alone served as the negative (solvent) control. The final DMSO concentration in all assays did not exceed 1% (v/v), a concentration that showed no detectable antimicrobial activity. Plates were incubated at 37 °C for 24 h, and antibacterial activity was assessed by measuring inhibition zone diameters (mm). All assays were conducted in triplicate.

#### 2.5.7 Minimum inhibitory and bactericidal concentrations (MIC/MBC)

The minimum inhibitory concentration (MIC) and minimum bactericidal concentration (MBC) of the Soxhlet extracts were determined by broth microdilution in Mueller–Hinton broth following Clinical and Laboratory Standards Institute (CLSI) guidelines (Wiegand et al., 2008). Two-fold serial dilutions ranging from 0.03125 to 8 mg/mL were prepared and tested against *S. aureus* ATCC 25923, *B. subtilis* ATCC 6633, *E. coli* ATCC 25922, and *P. aeruginosa* ATCC 27853, each adjusted to a final inoculum of approximately 1 × 10^6^ CFU/mL (Balouiri et al., 2016). Following incubation at 37 °C for 24 h, the MIC was defined as the lowest extract concentration yielding no visible bacterial growth. The MBC was subsequently determined by subculturing content from all growth-free wells onto Mueller–Hinton agar plates, with the lowest concentration producing no visible colonies after an additional 24 h of incubation recorded as the MBC. As several extracts were pigmented, viability in each well was confirmed by subculture onto agar rather than by turbidity alone, thereby minimising potential interference of extract colour with the MIC reading.

#### 2.5.8 Antifungal activity

The antifungal activity of *Cistus ladanifer* Soxhlet extracts was assessed using the direct contact method as previously described by Fandohan et al. (2004), with minor modifications. Four fungal strains were tested, comprising two dermatophytic fungi (*Trichophyton rubrum* and *Microsporum canis*) and two phytopathogenic fungi (*Botrytis cinerea* and *Fusarium oxysporum*). Each extract was incorporated at the tested concentration into molten potato dextrose agar (PDA), homogenized, and poured into sterile Petri dishes. Following solidification, a 6 mm mycelial plug obtained from a 5-day-old culture was centrally placed on each plate and incubated at 25 °C ± 2 °C for 5 days. Fungal radial growth was measured in two perpendicular directions, and antifungal activity was expressed as the percentage inhibition of mycelial growth relative to untreated controls. All assays were conducted in triplicate. Amphotericin B was included as a fungicidal positive control and DMSO, at the corresponding concentration, as the solvent control.

### 2.6 ADMET analysis

The four marker compounds selected for *in silico* evaluation—EOMCP, DHMAQ, esculin, and diosgenin—were prioritized from the GC-MS phytochemical profile according to three explicit criteria: (i) pharmacophoric diversity, in that the four compounds sample four distinct bioactive scaffold classes detected across the fractions (chromene/coumarin derivative, hydroxylated anthraquinone, coumarin glycoside, and steroidal sapogenin); (ii) literature-documented antimicrobial and antifungal relevance of these scaffold classes, in contrast to the numerically dominant constituents of the fractions (tetracosane, methyl docosanoate, myo-inositol, and D-glucuronic acid), which are epicuticular-wax, primary-metabolite, or cell-wall constituents lacking drug-like pharmacophores; and (iii) reliable spectral annotation, with NIST library match factors ≥ 87%. Accordingly, docking was applied to the structurally tractable, drug-like scaffolds and is intended as a hypothesis-generating prioritization step rather than a complete mechanistic account of the extracts’ activity.

The canonical SMILES strings of Ethyl 3-(2-oxo-7-methoxy-4-methylchromen-3-yl)propionate (EOMCP), 5,8-dihydroxy-6-methoxy-9,10-anthraquinone (DHMAQ), esculin, and diosgenin were retrieved from PubChem and used as input for SwissADME, pkCSM, and ADMET-AI (Pires et al., 2015; Daina et al., 2017; Swanson et al., 2024; Khibech et al., 2025). SwissADME and pkCSM were used to predict key physicochemical, drug-likeness, absorption, distribution, metabolism, excretion, and clearance parameters, while ADMET-AI was applied to estimate toxicity-related endpoints. This workflow served as an early *in silico* ADMET prioritization step and not as a substitute for experimental validation.

### 2.7 Molecular docking studies

**FIGURE 1:**
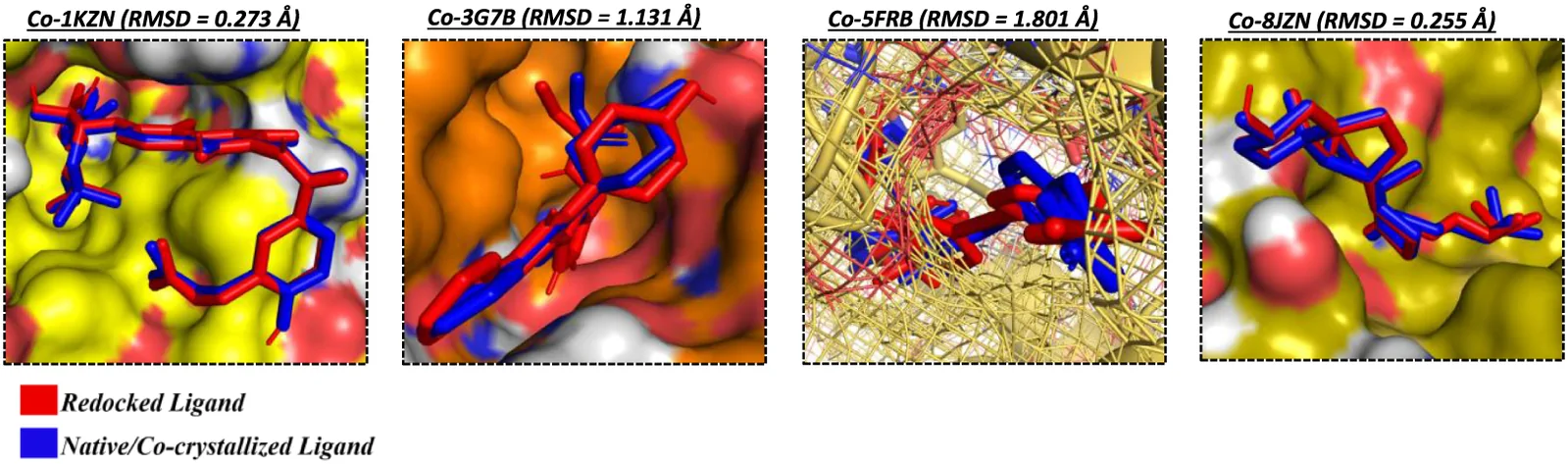
Redocking-based validation of the molecular docking protocol for 1KZN, 3G7B, 5FRB, and 8JZN. The redocked ligand is shown in red and the native/co-crystallized ligand in blue.

Molecular docking was performed using AutoDock Vina v1.2.0 implemented in PyRx v0.9 (Eberhardt et al., 2021; Trott and Olson, 2010; Khibech et al., 2026). The crystal structures of the selected antibacterial and antifungal target proteins (PDB IDs: 1KZN, 3G7B, 5FRB, and 8JZN) were prepared in AutoDockTools v1.5.7 by removing crystallographic water molecules and non-essential heteroatoms, adding polar hydrogens, assigning receptor charges, and converting the prepared structures into PDBQT format. When a crystallographic cofactor or prosthetic group formed part of the native binding environment, it was retained to preserve the structural integrity of the active pocket. The ligand structures of EOMCP, DHMAQ, Esculin, and Diosgenin were energy-minimized and converted into PDBQT format with appropriate torsion definitions. Docking reliability was assessed by redocking the native/co-crystallized ligands into their corresponding binding sites using an exhaustiveness value of 10. For 1KZN, the grid box was centered at x = 18.57, y = 30.77, z = 36.64 with dimensions x = 13.63, y = 16.75, z = 18.22 Å. For 3G7B, the grid box was centered at x = 50.87, y = −3.83, z = 19.91 with dimensions x = 14.14, y = 11.59, z = 10.17 Å. For 5FRB, the grid box was centered at x = 300.71, y = 10.77, z = 11.10 with dimensions x = 25.66, y = 7.76, z = 7.09 Å. For 8JZN, the grid box was centered at x = 178.44, y = 132.49, z = 165.34 with dimensions x = 16.46, y = 6.46, z = 11.22 Å. The redocking procedure reproduced the experimental ligand orientations with RMSD values of 0.273 Å for Co-1KZN, 1.131 Å for Co-3G7B, 1.801 Å for Co-5FRB, and 0.255 Å for Co-8JZN (Figure 1), supporting the suitability of the docking protocol for subsequent pose prediction and interaction analysis. Molecular visualization and figure preparation were carried out using PyMOL v2.5 and BIOVIA Discovery Studio 2024.

## 3 Statistical analysis

All experiments were performed using three independent biological replicates (i.e., three separate extractions and independently prepared assay cultures), each analyzed in technical triplicate, and results are expressed as mean ± standard deviation (SD). Statistical analyses were conducted using SPSS software (version 23.0; SPSS Inc., Chicago, IL, USA). Differences among the Soxhlet extracts were assessed by one-way analysis of variance (ANOVA) followed by Tukey’s *post hoc* test, with statistical significance set at p < 0.05. Prior to ANOVA, the homogeneity of variances was verified using Bartlett’s test and, given the small number of replicates per group (n = 3), the normality of residuals was assessed by graphical inspection. For the antifungal dataset, variance homogeneity was confirmed for each fungal species (Bartlett’s test, all p > 0.05).

Because Tukey’s HSD *post hoc* procedure intrinsically adjusts for all pairwise comparisons, the family-wise error rate was controlled at α = 0.05 without additional correction. For the antifungal dose–response data, a two-way ANOVA was additionally performed for each fungal species with extract type and concentration as fixed factors. Both main effects (extract: F (3,32) = 244–320; concentration: F (3,32) = 465–691; all p < 0.001) and their interaction (F (9,32) = 4.2–11.8; all p ≤ 0.001) were significant for every species, confirming that antifungal potency depended jointly on the solvent fraction and the applied concentration. Pairwise differences within each extract–species combination across concentrations are indicated by superscript letters in the corresponding table (Tukey’s HSD, p < 0.05).

The antifungal dose–response data were analyzed independently within each extract–species combination because the experimental design was established to compare concentration-dependent responses within individual treatments. Although factorial or mixed-effects models may provide additional insight into interaction effects among extract, concentration, and fungal species, such analyses were beyond the scope of the present experimental design and should be considered in future studies.

## 4 Results and discussion

### 4.1 Phytochemical content

**FIGURE 2:**
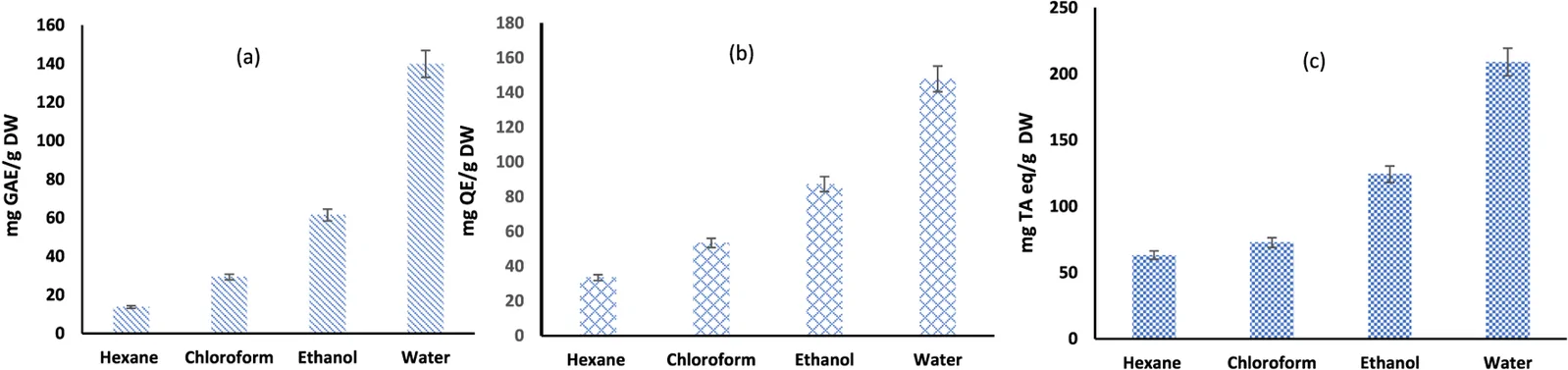
Polarity-dependent variation in total phenolic **(a)**, flavonoid **(b)**, and tannin **(c)** contents of *Cistus ladanifer* Soxhlet extracts.

The quantitative analysis of total phenolic, flavonoid, and tannin contents across the four Soxhlet extracts revealed a consistent polarity-dependent pattern, with polar extracts demonstrating significantly higher phytochemical accumulation compared to their apolar counterparts (Figure 2).

Total phenolic content (TPC) ranged from approximately 15 mg GAE/g DW in the hexane extract to 140 mg GAE/g DW in the aqueous extract, following the order: aqueous > ethanol > chloroform > hexane. A similar trend was observed for total flavonoid content (TFC), which ranged from approximately 28 mg QE/g DW (hexane) to 148 mg QE/g DW (aqueous extract). Total tannin content (TTC) exhibited the same polarity-dependent gradient, with values ranging from 62 mg TA eq/g DW in the hexane extract to approximately 210 mg TA eq/g DW in the aqueous extract, representing the highest phytochemical yield recorded across all three assays.

These findings collectively indicate that phenolic compounds, flavonoids, and tannins in *Cistus ladanifer* are predominantly hydrophilic in nature, exhibiting preferential solubility in polar solvents. The superior extraction efficiency of the aqueous and ethanolic fractions is consistent with the well-established physicochemical affinity of hydroxylated polyphenolic compounds for protic, hydrogen bond-donating solvents. Conversely, the markedly lower phytochemical yields observed in the hexane and chloroform fractions reflect the predominantly apolar character of these solvents, which selectively extract lipophilic constituents such as waxes, terpenoids, and fatty acids rather than polyphenolic metabolites (Alves-Ferreira et al., 2022; Sun et al., 2025).

The progressive increase in phytochemical content across the polarity gradient further underscores the critical role of solvent selection in optimizing the recovery of bioactive secondary metabolites from plant matrices, and supports the preferential use of polar solvents in the phytochemical characterization of *Cistus ladanifer*.

### 4.2 Chemical characterization

#### 4.2.1 FTIR spectroscopic analysis of soxhlet extracts

**FIGURE 3:**
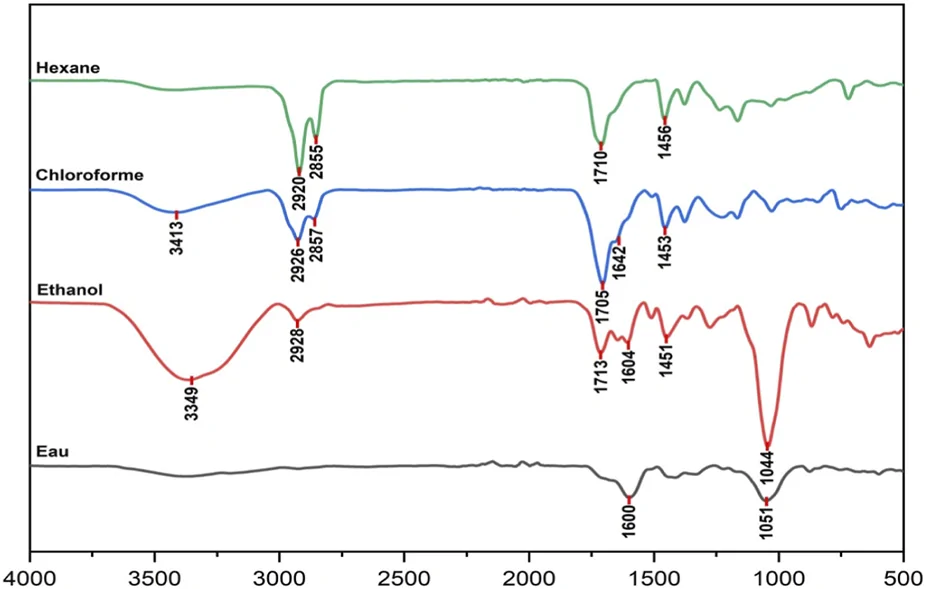
FTIR spectra of *Cistus ladanifer* Soxhlet fractions obtained with solvents of increasing polarity (hexane, chloroform, ethanol, and water).

The FTIR spectra of the four Soxhlet fractions obtained from *Cistus ladanifer* leaves are presented in Figure 3, with each fraction yielding a distinct spectral profile reflecting the polarity-dependent selectivity of the extraction solvents.

The hexane fraction was dominated by aliphatic C–H stretching vibrations at 2,920 and 2,855 cm^-1^, characteristic of lipids, hydrocarbons, and cuticular waxes (Šebek et al., 2011) alongside a prominent carbonyl band at 1710 cm^-1^ indicative of fatty acids or esters (Guillén and Cabo, 1997), confirming the lipophilic nature of this fraction. The chloroform fraction retained similar aliphatic absorptions at 2,926 and 2,857 cm^-1^, with an additional O–H stretching band at 3,413 cm^-1^ and an aromatic C=C vibration at 1,642 cm^-1^, consistent with the co-extraction of semi-polar terpenes and weakly polar phenolics (Mondal et al., 2019). The ethanol fraction was characterized by a broad O–H stretching absorption at 3,349 cm^-1^ and aromatic C=C vibrations at 1,604 cm^-1^, diagnostic of polyphenols and flavonoids (Bensemmane et al., 2021), with additional C–O stretching at 1,044 cm^-1^ attributable to glycosidic linkages (Guray-Deponthière, 2010). The aqueous fraction exhibited a dominant absorption at 1,600 cm^-1^ and C–O–C stretching bands at 1,051 and 1,044 cm^-1^, characteristic of polysaccharides and glycosidic compounds (Lin-Vien et al., 1991), with the near-complete absence of aliphatic absorptions confirming a matrix dominated by highly polar constituents.

Collectively, the FTIR data delineate a well-defined polarity gradient across the four fractions, providing compelling spectroscopic evidence that sequential Soxhlet extraction achieves effective and systematic chemical fractionation of the *Cistus ladanifer* leaf matrix.

#### 4.2.2 GC-MS analysis

The sequential Soxhlet extraction performed using solvents of increasing polarity—hexane, chloroform, ethanol, and water—yielded chemically distinct fractions whose GC-MS profiles strictly conform to the thermodynamic principle of *like dissolves like*, thereby providing a systematic and reproducible partitioning of the plant’s phytochemical constituents according to their polarity index, hydrogen-bonding capacity, and overall lipophilicity. The hexane fraction, representing the most apolar eluate (dielectric constant ε ≈ 1.9), was overwhelmingly dominated by long-chain *n*-alkanes, with tetracosane (C_24_H_50_) constituting the single most abundant compound across all fractions at 71.20%, followed by heptacosane (C_27_H_56_, 7.89%) and minor contributions from tricosane, pentacosane, and two octacosane isomers, collectively describing a characteristic epicuticular wax alkane assemblage biosynthetically derived from very long-chain fatty acid (VLCFA) decarboxylation pathways. The co-detection of fatty acid derivatives—including myristic acid, methyl linoleate, ethyl hexadecanoate, and methyl stearate—alongside the diterpenoids neophytadiene (0.30%) and phytol (0.19%) in this fraction is consistent with the well-established log P profiles of these compounds (log P > 4), whose hydrocarbon chain character supersedes the polarity contribution of their terminal functional groups, thus enabling their preferential partitioning into the apolar eluate. The chloroform fraction (ε ≈ 4.8), occupying the semi-polar intermediate of the solvent gradient, yielded the most chemically diverse profile, characterized by methyl docosanoate as the dominant constituent (23.00%), a long-chain fatty acid methyl ester whose amphiphilic character places it precisely within the solubility window of chloroform. Noteworthy in this fraction is the detection of ethyl 3-(2-oxo-7-methoxy-4-methylchromen-3-yl)propionate (10.74%), a functionalized chromene derivative whose planar aromatic ring system, coupled with moderate hydrogen-bond-accepting capacity, confers the intermediate polarity required for chloroform solubilization; the biological significance of this compound class is well documented, with coumarin and chromene scaffolds recognized for their broad-spectrum antimicrobial, antifungal, and anticoagulant activities. The monoterpenes camphene (1.61%) and β-pinene (1.03%), along with the kaurane-type diterpene 8,15-seco-8,16-*ent*-kaura-dien-15-ol (1.66%) and the anthraquinone derivative 5,8-dihydroxy-6-methoxy-9,10-anthraquinone (0.77%), further enrich the chloroform fraction with pharmacologically relevant scaffolds, the latter being of particular chemotaxonomic and biomedical interest given the well-established cytotoxic, laxative, and antimicrobial properties attributed to hydroxylated anthraquinones. The ethanol fraction (ε ≈ 24.6) marked a decisive shift in chemical character toward highly polar, polyhydroxylated metabolites, with myo-inositol emerging as the dominant compound at 41.60% — a cyclitol of fundamental physiological importance involved in membrane phospholipid biosynthesis, signal transduction, and osmotic stress response—followed by D-mannonic acid (23.41%), a sugar acid arising from the oxidation of mannose, whose detection at this magnitude reflects the significant saccharidic metabolic pool of the plant. Palmitic acid (6.43%), the most abundant saturated fatty acid in plant tissues, was exclusively recovered in the ethanol fraction rather than hexane or chloroform, an observation that may appear counterintuitive given its apolar hydrocarbon chain, but is fully rationalized by its occurrence in planta predominantly as a component of glycolipids, phospholipids, or ionically associated complexes that require a protic, polar solvent such as ethanol for effective disruption and solubilization. The additional detection in this fraction of quinic acid (4.48%), shikimic acid (4.33%), methyl α-D-glucofuranoside (3.37%), esculin (3.21%), β-galactofuranose (0.35%), cinnamic acid (0.12%), and diosgenin (1.68%) constructs a metabolic landscape spanning shikimate-pathway intermediates, glycosylated phenylpropanoids, and steroidal sapogenins, collectively underscoring ethanol’s unparalleled capacity as a broad-spectrum extractant for plant bioactives of pharmacological relevance. The aqueous fraction (ε ≈ 80.1), predictably, concentrated the most hydrophilic metabolites, with D-glucuronic acid dominating at 73.18% — a uronic acid integral to detoxification conjugation reactions and plant cell-wall pectin architecture—alongside malic acid (5.39%), quinic acid (2.91%), succinic acid (2.21%), and trace quantities of β-D-glucopyranose and oxalic acid (both reported as not resolved under the GC conditions employed), the latter pair attesting to the inherent limitation of GC-MS in quantifying highly polar, non-volatile carbohydrates without prior derivatization. Taken together, these results not only validate the chemical selectivity of sequential Soxhlet extraction as a preparative phytochemical tool but also delineate four pharmacologically meaningful chemical domains within the plant: an apolar lipid–wax phase harboring antifeedant and barrier-function metabolites, a semi-polar domain rich in terpenoids and phenylpropanoids with documented antimicrobial and cytotoxic activities, a polar alcohol-soluble fraction enriched in cyclitols and shikimate intermediates with antioxidant and anti-inflammatory potential, and a highly hydrophilic aqueous phase dominated by uronic acids and organic acids reflecting the plant’s primary metabolic and cell-wall carbohydrate pool.

It is important to emphasize that the bioactivity of the polar fractions reported in the following sections is not attributable to their numerically dominant GC-detectable constituents. myo-Inositol (41.60% of the ethanol fraction) and D-glucuronic acid (73.18% of the aqueous fraction) are primary metabolites and cell-wall precursors that are not established antimicrobial or antioxidant agents; their abundance in the GC-MS profile reflects their volatility and derivatization efficiency rather than their pharmacological weight. Rather, the observed activity tracks the polyphenolic pool quantified by the TPC, TFC, and TTC assays, which rises monotonically across the same polarity gradient (hexane → aqueous) as every bioactivity endpoint. The hydrolyzable tannins, ellagitannins, ellagic and gallic acid derivatives, and flavonoid glycosides characteristic of Cistus are non-volatile, thermolabile, and of high molecular weight, and are therefore not amenable to GC-MS even after silylation—which explains why they dominate the colorimetric quantification yet are absent from Table 1. Among the GC-detectable constituents, the chromene derivative (EOMCP, 10.74%) and the hydroxylated anthraquinone (DHMAQ, 0.77%) enriched in the chloroform fraction represent scaffolds with documented antimicrobial activity, consistent with that fraction’s intermediate potency. Definitive attribution of the activity to individual constituents would nonetheless require bioactivity-guided isolation.

**TABLE 1:** GC-MS identification and relative percentage composition of phytochemical constituents isolated from sequential Soxhlet extracts of *Cistus ladanifer* using solvents of increasing polarity.

Peak no.	RT (min)	Compound name	Molecular formula	Hex (%)	Chl (%)	EtOH (%)	AQ (%)	Match (%)
Long-chain Alkanes								
1	9.5	Tricosane	C_23_H_48_	0.28	—	—	—	93
2	46.17	Tetracosane	C_24_H_50_	71.20	—	—	—	97
3	38.78	Pentacosane	C_25_H_52_	0.42	—	—	—	95
4	47.61	Hexacosane	C_26_H_54_	—	2.51	—	—	96
5	42.59	Heptacosane	C_27_H_56_	7.89	0.80	—	—	94
6	44.26	Octacosane	C_28_H_58_	0.76	—	—	—	91
7	49.23	Octacosane (isomer)	C_28_H_58_	0.53	—	—	—	90
8	45.14	Tritriacontane	C_33_H_68_	—	1.12	—	—	92
Fatty acids and derivatives								
9	21.24	Myristic acid	C_14_H_28_O_2_	0.22	—	—	—	95
10	30.35	Methyl linoleate	C_19_H_34_O_2_	0.89	—	—	—	93
11	26.37	Ethyl hexadecanoate	C_18_H_36_O_2_	0.81	—	—	—	94
12	31.08	Methyl stearate	C_19_H_38_O_2_	0.57	1.67	—	—	96
13	40.25	Methyl docosanoate	C_23_H_46_O_2_	—	23.00	—	—	97
14	24.62	Palmitic acid	C_16_H_32_O_2_	—	—	6.43	—	98
Monoterpenes								
15	30.47	Camphene	C_10_H_16_	—	1.61	—	—	95
16	25.27	β-Pinene	C_10_H_16_	—	1.03	—	—	93
Diterpenes								
17	26.36	Neophytadiene	C_20_H_38_	0.30	1.12	—	—	91
18	30.9	Phytol	C_20_H_40_O	0.19	—	—	—	90
19	35.67	8,15-Seco-8,16-ent-kaura-dien-15-ol	C_20_H_32_O	—	1.66	—	—	88
Phenolic and chromene derivatives								
20	24.4	Cinnamic acid	C_9_H_8_O_2_	—	—	0.12	—	94
21	40.72	Ethyl 3-(2-oxo-7-methoxy-4-methylchromen-3-yl)propionate	C_16_H_18_O_5_	—	10.74	—	—	89
Anthraquinones								
22	32.07	5,8-Dihydroxy-6-methoxy-9,10-anthraquinone	C_15_H_10_O_5_	—	0.77	—	—	87
Cyclitols								
23	24.54	Myo-inositol	C_6_H_12_O_6_	—	—	41.60	—	98
Sugar acids and uronic acids								
24	25.63	D-mannonic acid	C_6_H_12_O_7_	—	—	23.41	—	92
25	24.23	D-glucuronic acid	C_6_H_10_O_7_	—	—	—	73.18	96
Organic acids								
26	25.37	Quinic acid	C_7_H_12_O_6_	—	—	4.48	2.91	97
27	24.46	Shikimic acid	C_7_H_10_O_5_	—	—	4.33	—	95
28	15.03	Malic acid	C_4_H_6_O_5_	—	—	—	5.39	96
29	9.41	Succinic acid	C_4_H_6_O_4_	—	—	—	2.21	94
30	17.29	Oxalic acid	C_2_H_2_O_4_	—	—	—	1.51	85
Carbohydrates								
31	24.11	Methyl α-D-glucofuranoside	C_7_H_14_O_6_	—	—	3.37	—	93
32	24.25	β-Galactofuranose	C_6_H_12_O_6_	—	—	0.35	—	91
33	23.99	β-D-Glucopyranose	C_6_H_12_O_6_	—	—	—	3.81	88
Glycosides and coumarins								
34	40.52	Esculin	C_15_H_16_O_9_	—	—	3.21	—	90
Steroidal compounds								
35	36.62	Diosgenin	C_27_H_42_O_3_	—	—	1.68	—	92

Consistent with this, all GC-MS assignments reported here should be regarded as tentatively identified: they rest on NIST spectral matching and retention behaviour rather than on co-injection of authentic standards, and the relative percentages refer to GC-amenable, derivatizable constituents only. In particular, the hydrolyzable tannins, ellagic acid derivatives, and flavonoid glycosides invoked in the antioxidant and antimicrobial discussion are inferred from the colorimetric phytochemical assays and from prior reports on Cistus rather than detected by GC-MS; their confirmation would require complementary LC-MS/MS or HPLC-DAD analysis.

### 4.3 Antioxidant and antimicrobial activities

#### 4.3.1 Antioxidant activity

**FIGURE 4:**
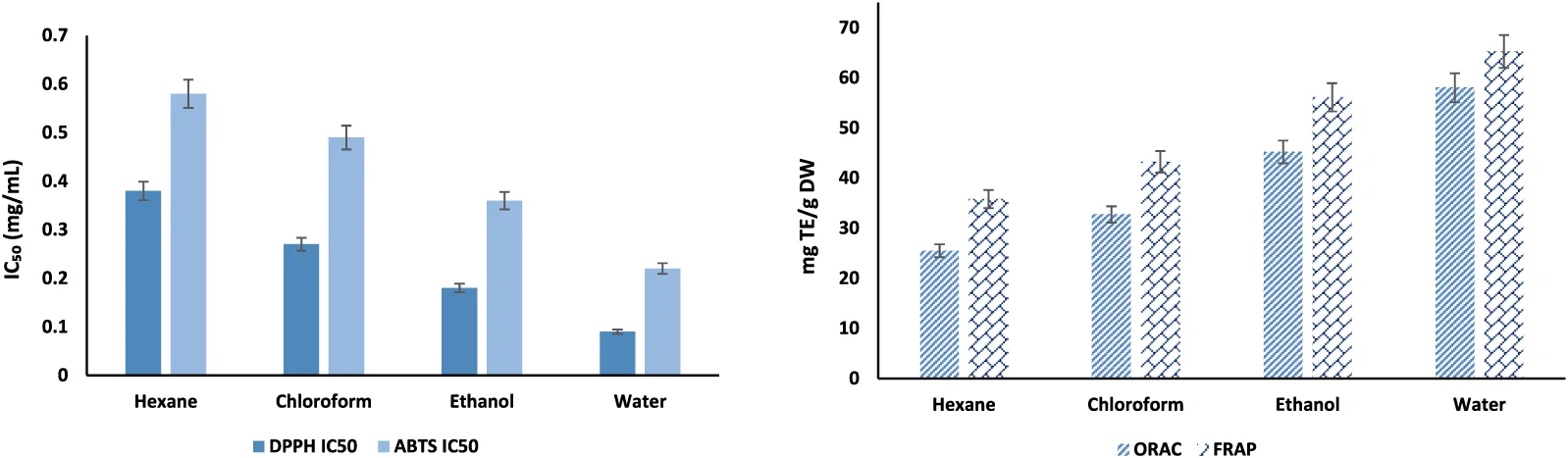
Radical scavenging activity (DPPH and ABTS IC_50_, mg/mL) and antioxidant capacity (ORAC and FRAP, mg TE/g DW) of *Cistus ladanifer* leaf Soxhlet fractions as a function of solvent polarity.

The antioxidant activity of the four *Cistus ladanifer* Soxhlet fractions, evaluated by four complementary assays covering two distinct mechanistic pathways, is presented in Figure 4. A consistent and statistically significant polarity-dependent gradient was observed across all assays, with antioxidant potency increasing progressively from the hexane to the aqueous fraction—a pattern that will be further corroborated by the antibacterial and antifungal bioactivity data presented in Sections 3.3 and 3.4 below, and that collectively points to the polar fraction of *C. ladanifer* as the primary reservoir of bioactive secondary metabolites.

For the DPPH radical scavenging assay, IC_50_ values decreased from 0.38 ± 0.005 mg/mL (hexane) to 0.27 ± 0.003 mg/mL (chloroform), 0.18 ± 0.002 mg/mL (ethanol), and 0.09 ± 0.001 mg/mL (aqueous fraction), representing a 4.2-fold enhancement in radical scavenging potency across the full polarity range. A parallel trend was observed for the ABTS radical cation decolorization assay, with IC_50_ values declining from 0.58 ± 0.03 mg/mL (hexane) to 0.22 ± 0.03 mg/mL (aqueous fraction), corresponding to a 2.6-fold increase in potency. The ORAC values—which measure the capacity to quench peroxyl radicals through hydrogen atom transfer (HAT) — increased from 25.5 ± 0.5 mg TE/g DW (hexane) to 32.73 ± 0.6, 45.21 ± 0.4, and 58.0 ± 0.5 mg TE/g DW for the chloroform, ethanolic, and aqueous fractions, respectively, while FRAP values followed an analogous ascending gradient from 35.8 ± 0.2 mg TE/g DW (hexane) to 65.25 ± 0.2 mg TE/g DW (aqueous fraction). The concordance of all four assays in identifying the aqueous fraction as the most potent is of particular mechanistic significance: DPPH and ABTS primarily probe single electron transfer (SET) mechanisms, while ORAC is exclusively sensitive to HAT-mediated radical quenching, and FRAP measures reductive capacity through ferric ion reduction. The fact that the aqueous fraction consistently outperforms all other fractions across both mechanistic classes indicates that its antioxidant constituents operate through genuinely multi-mechanistic pathways rather than through a single dominant reaction type, a hallmark of complex polyphenolic mixtures and a feature that substantially strengthens their biological relevance in oxidative stress contexts (Prior et al., 2003; Huang et al., 2005).

The polarity-activity relationship observed across all four assays is mechanistically consistent with the established phytochemical profile of *Cistus ladanifer*, a species whose antioxidant activity has been attributed primarily to a diverse array of polar phenolic compounds including hydrolyzable tannins—notably punicalagin, punicalin, and casuariin—ellagic acid and its glycoside derivatives, gallic acid, quercetin-3-O-glucoside, kaempferol-3-O-rutinoside, and a range of hydroxycinnamic acid esters (Barrajón-Catalán et al., 2011). These compound classes share the capacity to donate hydrogen atoms or electrons to radical species through their multiple hydroxyl substituents on aromatic rings, with their antioxidant potency further enhanced by intramolecular hydrogen bonding, resonance stabilization of the resulting phenoxyl radicals, and in the case of ellagitannins, the additional contribution of galloyl ester groups that multiply the number of reactive hydroxyl positions per molecule. Their preferential extraction in hot aqueous media—either through direct solubilization or through the thermally driven hydrolysis of ester-linked precursors—accounts for the superior performance of the aqueous fraction and is consistent with the ethnobotanical tradition of preparing *C. ladanifer* as a hot infusion or decoction in Moroccan and Mediterranean folk medicine for its anti-inflammatory and antioxidant properties.

The meaningful antioxidant activity recorded for the hexane fraction across all four assays—despite its non-polar composition—is noteworthy and warrants specific discussion. Unlike the near-absent antibacterial activity of this fraction against Gram-negative pathogens, the hexane extract exhibited detectable DPPH (IC_50_ = 0.38 mg/mL) and ABTS (IC_50_ = 0.58 mg/mL) scavenging activity, as well as measurable ORAC (25.5 mg TE/g DW) and FRAP (35.8 mg TE/g DW) values. This activity is attributable to the labdane-type diterpenes characteristic of *C. ladanifer*—in particular labdanolic acid, cistadiol, and related compounds—which contain hydrogen-donating hydroxyl and carboxylic acid functional groups capable of participating in radical scavenging reactions despite their overall lipophilic character. Additionally, certain terpenoid quinones and tocopherol-related compounds that may be co-extracted in the hexane fraction are established lipophilic antioxidants whose contribution to peroxyl radical quenching in the ORAC assay—conducted in an aqueous-organic medium—can be non-negligible. The antioxidant activity of the hexane fraction may be of particular relevance in lipophilic biological compartments such as cell membranes, where polar polyphenolics are less effective, suggesting a potentially complementary mechanistic profile between the non-polar and polar fractions of this plant.

The ABTS IC_50_ values were consistently higher than the corresponding DPPH IC_50_ values across all fractions, a relationship that has been reported in multiple studies on polyphenol-rich plant extracts and reflects the differential reactivity of the ABTS radical cation—a larger, more stable chromophore—compared to the smaller, more electrophilic DPPH radical, with which phenolic compounds react more readily through both HAT and SET pathways. Conversely, the ORAC values systematically exceeded the FRAP values expressed in Trolox equivalents for all fractions, consistent with the greater sensitivity of the ORAC assay to compounds with high HAT capacity—particularly the ellagitannins and gallic acid derivatives enriched in the polar fractions—relative to the FRAP assay, which specifically measures reductive electron-donating capacity in a low-pH buffer and may underestimate the contribution of slower-reacting antioxidants present in the chloroform and ethanolic fractions. These inter-assay discrepancies underscore the methodological imperative of employing multiple complementary assays for the comprehensive characterization of plant extract antioxidant activity, as no single assay captures the full mechanistic spectrum of radical scavenging and reducing capacity.

The DPPH IC_50_ value of 0.09 ± 0.001 mg/mL recorded for the aqueous extract and the FRAP value of 65.25 ± 0.2 mg TE/g DW are comparable to or exceed those reported for well-characterized antioxidant-rich plant extracts including green tea (*Camellia sinensis*), pomegranate peel (*Punica granatum*), and rosemary (*Rosmarinus officinalis*) under equivalent experimental conditions, positioning the aqueous Soxhlet fraction of *C. ladanifer* among the most potent natural antioxidant sources documented to date and providing a strong scientific basis for its valorization in the development of natural antioxidant ingredients for pharmaceutical, nutraceutical, and cosmetic applications. Future investigations should focus on the isolation and structural identification of the individual antioxidant constituents responsible for the observed activity through bioactivity-guided fractionation, the determination of structure-activity relationships across the identified polyphenolic series, and the evaluation of antioxidant efficacy in biologically relevant cellular oxidative stress models—including lipid peroxidation inhibition assays and cellular reactive oxygen species (ROS) quantification—to bridge the gap between *in vitro* antioxidant potential and *in vivo* therapeutic relevance.

#### 4.3.2 Antibacterial activity

**FIGURE 5:**
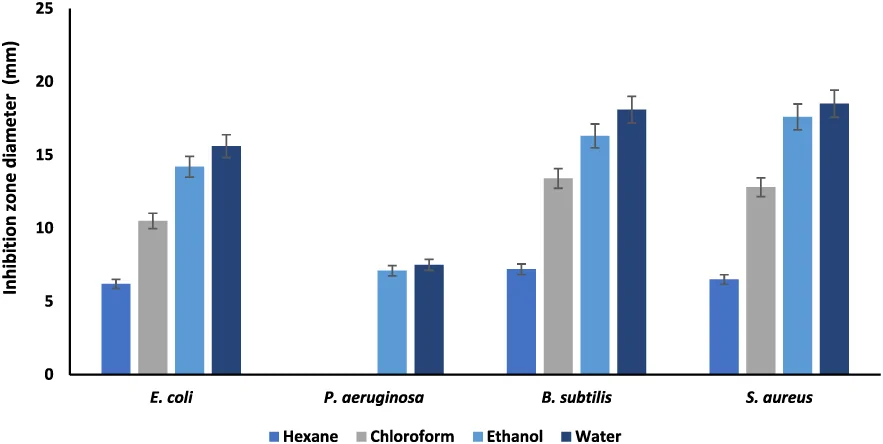
Antibacterial activity of *Cistus ladanifer* leaf soxhlet extracts as a function of solvent polarity.

The antimicrobial activity of the four solvent extracts against the tested bacterial strains, expressed as inhibition zone diameters, is presented in Figure 5. All extracts exhibited variable degrees of antibacterial activity depending on both the nature of the solvent and the bacterial species tested, and a clear polarity-dependent gradient was observed throughout the dataset, with inhibition zone diameters increasing progressively from the hexane to the aqueous extract. The hexane extract demonstrated only weak inhibitory effects against *Escherichia coli* (6.2 ± 0.5 mm), *Bacillus subtilis* (7.2 ± 0.8 mm), and *Staphylococcus aureus* (6.5 ± 0.6 mm), and was entirely devoid of activity against *Pseudomonas aeruginosa*. The chloroform extract showed moderate activity against *E. coli* (10.5 ± 0.7 mm), *B. subtilis* (13.4 ± 1.0 mm), and *S. aureus* (12.8 ± 0.9 mm), but similarly produced no detectable inhibition zone against *P. aeruginosa*. The ethanolic extract ranked second in overall potency, recording inhibition zones of 14.2 ± 1.1 mm, 7.1 ± 0.8 mm, 16.3 ± 1.3 mm, and 17.6 ± 1.2 mm against *E. coli*, *P. aeruginosa*, *B. subtilis*, and *S. aureus*, respectively, while the aqueous extract demonstrated the highest inhibitory activity across all susceptible strains, with inhibition zone diameters of 15.6 ± 0.9 mm, 7.5 ± 0.6 mm, 18.1 ± 1.0 mm, and 18.5 ± 0.8 mm against the same organisms. In addition to this solvent-dependent pattern, a consistent differential in susceptibility between Gram-positive and Gram-negative bacteria was observed across all extracts, with *B. subtilis* and *S. aureus* displaying systematically larger inhibition zones than *E. coli* and *P. aeruginosa*.

Because agar-based diffusion assays can be influenced by the differential diffusion of analytes of varying polarity and molecular weight, several measures were taken to limit diffusion bias, and the interpretation of these data does not rest on the diffusion assay alone. First, the inoculum was standardized to the 0.5 McFarland turbidity standard, a fixed extract volume (25 µL) was applied to uniformly sized 6 mm wells, and solvent-only negative controls were included on every plate. Second, and more importantly, the diffusion results were independently corroborated by broth microdilution (MIC/MBC), a quantitative method that is not subject to diffusion artefacts and that reproduced the same polarity-dependent susceptibility ranking. This orthogonal agreement strengthens confidence in the antibacterial gradient reported here. We nonetheless recognize that confirmatory mechanistic assays—including time–kill kinetics, propidium iodide uptake, and ATP-leakage measurements—would be required to substantiate the membrane-level interpretations proposed below; these are identified as priority follow-up work but were beyond the scope of the present study.

The progressive increase in antimicrobial activity along the polarity gradient—hexane < chloroform < ethanol < water—strongly suggests that the principal antimicrobial constituents of this plant are polar in nature. Non-polar solvents such as hexane selectively extract lipophilic compounds including hydrocarbons, waxes, fixed oils, and certain terpenes, which are generally recognized as poor inhibitors of bacterial growth at the concentrations achievable in well diffusion systems. The moderate activity of the chloroform fraction may reflect the partial extraction of moderately polar compounds such as flavonoid aglycones, coumarins, or lipophilic alkaloids, which possess documented membrane-disrupting properties but are present at suboptimal concentrations relative to the more polar fractions. The strong activity exhibited by the ethanolic and aqueous extracts is consistent with the preferential enrichment of well-established polar antimicrobial compound classes—including phenolic acids, flavonoid glycosides, condensed and hydrolyzable tannins, and polar alkaloids. These compound classes have been reported to exhibit antibacterial activity through mechanisms that may include membrane perturbation, interference with cell wall biosynthesis, metal-ion chelation, and inhibition of nucleic acid or protein synthesis. However, the present study did not directly investigate these mechanisms, and they should therefore be regarded as plausible hypotheses requiring experimental validation. These mechanistic considerations underscore the relevance of optimizing solvent polarity as a primary variable in any bioactivity-guided fractionation strategy applied to this plant material.

The superior activity of the aqueous extract over the ethanolic fraction, while counter-intuitive given that ethanol is often regarded as a more efficient universal extraction solvent, is nonetheless a reproducible finding in the phytochemical literature and can be interpreted on several grounds. Hot aqueous extraction, as employed in traditional decoctions, is capable of hydrolyzing ester- and glycosidic-linked phenolic conjugates that remain intact under ethanolic extraction conditions, thereby releasing pharmacologically active aglycones and low-molecular-weight phenolic acids into solution. Furthermore, certain high-molecular-weight tannins and mucilaginous polysaccharides with reported antibacterial activity are preferentially solubilized in water rather than in aqueous-organic mixtures, and the hydrogen-bonding capacity of water may additionally facilitate the solubilization of protonated alkaloids and charged phenolic species that exhibit reduced solubility in ethanol. This interpretation finds support in analogous findings reported for aqueous extracts of *Punica granatum*, *Lawsonia inermis*, and several *Acacia* species, where water fractions consistently outperformed ethanolic ones in well diffusion assays against *Staphylococcus aureus*. To definitively confirm this interpretation in the context of the present study, quantitative phytochemical analyses—including total phenolic content (TPC), total flavonoid content (TFC), and condensed tannin quantification—should be conducted across all fractions to establish a direct and statistically supported correlation between polar compound concentration and observed antibacterial potency.

The differential susceptibility observed between Gram-positive and Gram-negative bacteria is mechanistically consistent with the fundamental architectural differences between these two groups. Gram-positive bacteria, including *B. subtilis* and *S. aureus*, are bounded by a single cytoplasmic phospholipid bilayer surrounded by a thick but structurally open peptidoglycan network that does not constitute an effective permeability barrier for most plant-derived polar compounds. Gram-negative bacteria, in contrast, possess an additional outer membrane whose outer leaflet is composed predominantly of lipopolysaccharide (LPS), a molecule that confers marked resistance to the passive diffusion of hydrophilic antimicrobials by creating a highly ordered, low-permeability surface. This outer membrane, acting in concert with the narrow substrate specificity of porins and the activity of constitutively expressed efflux pump systems, significantly reduces the intracellular accumulation of bioactive molecules and has been identified as the primary determinant of intrinsic resistance in Gram-negative pathogens. The higher inhibition zones consistently recorded for *S. aureus* relative to *B. subtilis* across all active extracts may further reflect intrinsic differences in cell wall thickness, teichoic acid composition, or membrane fluidity between the two species, and warrants dedicated mechanistic investigation. The complete absence of inhibitory activity of the hexane and chloroform extracts against *P. aeruginosa* corroborates and extends this interpretation. *P. aeruginosa* is widely recognized, on the basis of established microbiology, as one of the most intrinsically resistant bacterial species to plant-derived antimicrobials, a phenotype attributable to its exceptionally low outer-membrane permeability—estimated at 1%–8% of that of *E. coli*—the constitutive overexpression of broad-spectrum efflux pump systems including MexAB-OprM and MexCD-OprJ, and the relative paucity of functional non-specific porins in its outer membrane. Non-polar compounds extracted by hexane and chloroform, lacking the structural features necessary to disrupt the LPS layer or to evade efflux mechanisms, predictably fail to achieve minimum inhibitory concentrations against this organism. The residual, though weak, sensitivity of *P. aeruginosa* to the ethanol and water extracts (7.1 and 7.5 mm, respectively) nevertheless indicates that certain polar constituents of this plant possess the physicochemical properties required to at least partially overcome the permeability barrier of this highly resistant pathogen, and these compounds represent priority targets for isolation and structural characterization.

Taken together, the well diffusion results provide a clear polarity-dependent antibacterial profile across both Gram-positive and Gram-negative organisms. The minimum inhibitory and bactericidal concentrations determined by broth microdilution are presented in the following section.

The minimum inhibitory concentration (MIC), minimum bactericidal concentration (MBC), and MBC/MIC ratios of the four *Cistus ladanifer* Soxhlet extracts against the tested bacterial strains are presented in Table 2. The results revealed considerable variation in antibacterial potency across both solvent systems and bacterial species, and demonstrated a clear and consistent inverse relationship between solvent polarity and MIC values that fully corroborates the inhibition zone gradient previously established by well diffusion assay. The aqueous extract exhibited the lowest MIC values overall, recording 0.5 mg/mL against *Escherichia coli*, 4 mg/mL against *Pseudomonas aeruginosa*, and 0.25 mg/mL against both *Bacillus subtilis* and *Staphylococcus aureus*, confirming it as the most potent fraction. The ethanolic extract ranked second in overall potency, with MIC values of 1 mg/mL, 8 mg/mL, 0.5 mg/mL, and 0.5 mg/mL against *E. coli*, *P. aeruginosa*, *B. subtilis*, and *S. aureus*, respectively. The chloroform extract demonstrated moderate potency, with MIC values of 4 mg/mL against *E. coli*, 1 mg/mL against *B. subtilis*, and 2 mg/mL against *S. aureus*, while remaining entirely inactive against *P. aeruginosa* (ND). The hexane extract was the least potent fraction, recording MIC values of 8 mg/mL against *E. coli* and *S. aureus* and 4 mg/mL against *B. subtilis*, with no detectable inhibitory activity against *P. aeruginosa* (ND). MBC values were consistently two-to fourfold higher than their corresponding MIC values across all extract-strain combinations, and MBC/MIC ratios ranged from 2 to 4 depending on the extract and the bacterial species tested, with ratios of 2 recorded uniformly for the ethanol and water extracts and ratios of 4 observed for the hexane and chloroform extracts against *E. coli*.

**TABLE 2:** Minimum inhibitory concentration (MIC), minimum bactericidal concentration (MBC), and MBC/MIC ratios of *Cistus ladanifer* leaf soxhlet extracts against selected gram-positive and gram-negative bacterial strains.

Extracts	Inhibition parameters	Bacteria strains			
		*E. coli*	*P. aeruginosa*	*B. subtilis*	*S. aureus*
Hexane	MIC	8	ND	4	8
	MBC	32	ND	8	16
	MBC/MIC	4	ND	2	2
Chloroform	MIC	4	ND	1	2
	MBC	16	ND	2	4
	MBC/MIC	4	ND	2	2
Ethanol	MIC	1	8	0.5	0.5
	MBC	2	16	1	1
	MBC/MIC	2	2	2	2
Water	MIC	0.5	4	0.25	0.25
	MBC	1	8	0.5	0.5
	MBC/MIC	2	2	2	2

MIC, minimum inhibitory concentration; MBC, minimum bactericidal concentration; ND, Not determined (no inhibition zone detected in well diffusion assay).

The inverse relationship between solvent polarity and MIC values—with the aqueous extract outperforming the hexane extract by factors of 16–32 against Gram-positive strains—is in full agreement with the well diffusion data and strongly supports the hypothesis that the principal bioactive constituents of this plant are polar in nature. This polarity-activity relationship is well documented for Mediterranean shrubs of the Cistaceae family, which are characteristically rich in hydrolyzable tannins, ellagic acid derivatives, flavonoid glycosides, and hydroxycinnamic acids—compound classes whose optimal extraction requires polar aqueous or hydroalcoholic media and whose antibacterial mechanisms involve membrane permeabilization, metal ion chelation, and inhibition of key bacterial enzyme systems including DNA gyrase and the fatty acid synthase complex (FAS II) (Cowan, 1999; Cushnie and Lamb, 2005; Scalbert, 1991). The markedly higher potency of the aqueous extract relative to the ethanolic fraction—despite ethanol being widely regarded as a superior universal extraction solvent—may be explained by the preferential solubilization in hot water of high-molecular-weight ellagitannins and gallic acid conjugates, which are among the principal antimicrobial constituents of *Cistus* species (Attaguile et al., 2000; Smiljkovic et al., 2017), as well as by the hydrolysis of ester-linked phenolic conjugates under aqueous extraction conditions, a process that releases pharmacologically active aglycones and low-molecular-weight phenolic acids unavailable in ethanolic extracts. These mechanistic considerations underscore the relevance of aqueous extraction as a primary preparative strategy for this plant material, and are consistent with the traditional use of *C. ladanifer* as an aqueous infusion or decoction in Moroccan and Mediterranean ethnomedicine.

The complete absence of measurable MIC and MBC values for the hexane and chloroform extracts against *P. aeruginosa* is mechanistically consistent with the intrinsic resistance profile of this organism and validates the internal coherence of the full dataset. *P. aeruginosa* possesses one of the most impermeable outer membranes among clinically relevant Gram-negative pathogens, with an intrinsic permeability estimated at 1%–8% of that of *E. coli*, attributable to its highly ordered lipopolysaccharide (LPS) outer leaflet, restricted expression of non-specific porins (OprD), and constitutive overexpression of the MexAB-OprM and MexCD-OprJ efflux pump systems (Hancock and Speert, 2000; Nikaido, 2003). Non-polar compounds extracted by hexane—principally terpenoids, fatty acids, and waxes—lack the amphiphilic character required to intercalate into and disrupt the LPS bilayer, and are efficiently expelled from the periplasmic space by the broad-substrate efflux mechanisms of this organism before intracellular concentrations sufficient for growth inhibition can be achieved. The chloroform fraction, while capable of extracting moderately polar compounds such as flavonoid aglycones and lipophilic alkaloids with partial membrane affinity, similarly fails to reach the inhibitory threshold against *P. aeruginosa*, in full agreement with the absence of an inhibition zone for this fraction in the well diffusion assay. The residual but quantifiable sensitivity of *P. aeruginosa* to the ethanolic (MIC = 8 mg/mL) and aqueous (MIC = 4 mg/mL) extracts suggests that certain highly polar constituents of *C. ladanifer*—potentially including gallic acid, ellagic acid, or polar flavonoid derivatives—possess the amphiphilic or membrane-active structural features necessary to at least partially overcome the permeability barrier of this pathogen, likely through LPS displacement or outer membrane destabilization, mechanisms that have been documented for plant-derived polyphenols at elevated concentrations (Burt, 2004).

The consistently lower MIC and MBC values recorded for Gram-positive bacteria (*B. subtilis* and *S. aureus*) relative to Gram-negative organisms across all active extracts further reinforces the structural basis of the observed susceptibility differential. The absence of an outer membrane in Gram-positive species allows plant-derived polar antimicrobials direct access to the cytoplasmic membrane and peptidoglycan layer, enabling them to exert their bactericidal effects—membrane depolarization, pore formation, and inhibition of cell wall biosynthetic enzymes—at significantly lower concentrations than those required to overcome the dual permeability barriers of Gram-negative bacteria (Nikaido, 2003). The slightly lower MIC values recorded for *B. subtilis* relative to *S. aureus* in the hexane and chloroform fractions (4 vs. 8 mg/mL and 1 vs. 2 mg/mL, respectively) may reflect intrinsic differences in membrane composition, teichoic acid density, or the expression of resistance determinants between the two species; however, both organisms displayed equal susceptibility to the more potent ethanolic and aqueous extracts (MIC = 0.5 and 0.25 mg/mL, respectively), indicating that at sufficiently high polar compound concentrations, these species-level structural differences are functionally overcome and no longer constitute a meaningful barrier to bactericidal action.

The MBC/MIC ratios constitute perhaps the most mechanistically informative dimension of the dataset. By established convention, a ratio of ≤4 is indicative of bactericidal activity, while ratios >4 denote a bacteriostatic mode of action (Pankey and Sabath, 2004). In the present study, all MBC/MIC ratios fell within the range of 2 to 4, classifying all active fractions as bactericidal against their respective susceptible organisms across the full concentration range tested. The uniform ratio of 2 observed for the ethanol and water extracts against all susceptible strains indicates a narrow concentration window separating complete growth inhibition from complete bacterial killing, a hallmark of compounds acting through irreversible disruption of the bacterial cytoplasmic membrane or covalent modification of essential enzymatic targets — mechanisms characteristic of tannins and polyphenolic acids, which constitute the dominant antimicrobial classes of polar *Cistus* extracts (Scalbert, 1991). The higher MBC/MIC ratio of 4 observed for the hexane and chloroform extracts against *E. coli*—compared to a ratio of 2 against Gram-positive strains—reflects the greater energetic and concentration requirements for achieving complete bacterial killing in organisms protected by a dual membrane architecture: while partial disruption of the LPS outer membrane can suppress growth at moderate concentrations, irreversible lethal damage to the cytoplasmic membrane demands higher concentrations capable of penetrating both barriers sequentially, a challenge absent in Gram-positive species. This differential is of clinical relevance, as bactericidal agents are generally preferred over bacteriostatic ones in the management of immunocompromised patients and systemic infections, and the strict bactericidal classification of the polar fractions against all susceptible organisms—including the clinically critical pathogen *S. aureus*—substantially strengthens the therapeutic significance of these extracts.

Considered in their totality, the MIC, MBC, and MBC/MIC data presented in this study establish that the polar fractions of *C. ladanifer*, and in particular the aqueous extract, possess quantitatively significant, mechanistically coherent, and bactericidal antibacterial activity against a clinically representative panel of both Gram-positive and Gram-negative bacterial species. The MIC values of 0.25 mg/mL recorded for the aqueous extract against *B. subtilis* and *S. aureus* are comparable to those reported for purified plant-derived antimicrobials such as thymol and carvacrol, and fall within the range of biological relevance for the development of natural antibacterial agents (Burt, 2004). Future investigations should undertake bioactivity-guided fractionation of the aqueous and ethanolic extracts coupled with time-kill kinetic assays, membrane integrity studies (propidium iodide uptake, ATP leakage measurements), and molecular docking analyses to identify the specific compounds responsible for the observed bactericidal effects and to elucidate their precise mechanisms of action at the molecular level.

#### 4.3.3 Antifungal activity

The antifungal activity of the four *Cistus ladanifer* Soxhlet extracts against the tested fungal strains, expressed as mycelial growth inhibition percentages at increasing concentrations, is presented in Table 3. All extracts demonstrated concentration-dependent inhibitory activity against the four tested species, with inhibition values ranging from 5.0% ± 1.0% (hexane extract, 0.5 mg/mL, *Fusarium oxysporum*) to 86.0% ± 3.0% (aqueous extract, 4.0 mg/mL, *Trichophyton rubrum*), and two overarching patterns emerged consistently across the entire dataset: a clear polarity-dependent gradient in antifungal potency and a systematic differential in susceptibility between dermatophytic and phytopathogenic fungal species.

**TABLE 3:** Antifungal activity of Cistus ladanifer leaf soxhlet extracts: Effect of solvent polarity on mycelial growth inhibition (%) against dermatophytic and phytopathogenic fungal strains.

Extracts	Concentration (mg/mL)	Mycelial inhibition (%)			
		*T. rubrum*	*M. canis*	*B. cinerea*	*F. oxysporum*
Hexane	0.5	12.0 ± 1.5 ^d^	10.0 ± 1.0 ^d^	6.0 ± 1.0 ^d^	5.0 ± 1.0 ^d^
	1.0	22.0 ± 2.0 ^c^	18.0 ± 2.0 ^c^	12.0 ± 1.5 ^c^	10.0 ± 1.0 ^c^
	2.0	35.0 ± 3.0 ^b^	30.0 ± 2.0 ^b^	20.0 ± 2.0 ^b^	16.0 ± 2.0 ^b^
	4.0	48.0 ± 3.0 ^a^	42.0 ± 3.0 ^a^	30.0 ± 2.0 ^a^	25.0 ± 2.0 ^a^
Chloroform	0.5	20.0 ± 2.0 ^d^	17.0 ± 2.0 ^d^	12.0 ± 1.5 ^d^	10.0 ± 1.0 ^d^
	1.0	35.0 ± 3.0 ^c^	30.0 ± 2.0 ^c^	22.0 ± 2.0 ^c^	18.0 ± 2.0 ^c^
	2.0	52.0 ± 3.0 ^b^	46.0 ± 3.0 ^b^	35.0 ± 3.0 ^b^	28.0 ± 2.0 ^b^
	4.0	68.0 ± 4.0 ^a^	62.0 ± 3.0 ^a^	48.0 ± 3.0 ^a^	40.0 ± 3.0 ^a^
Ethanol	0.5	28.0 ± 2.0 ^d^	24.0 ± 2.0 ^d^	18.0 ± 2.0 ^d^	14.0 ± 1.5 ^d^
	1.0	48.0 ± 3.0 ^c^	42.0 ± 3.0 ^c^	32.0 ± 3.0 ^c^	26.0 ± 2.0 ^c^
	2.0	65.0 ± 4.0 ^b^	60.0 ± 3.0 ^b^	48.0 ± 3.0 ^b^	40.0 ± 3.0 ^b^
	4.0	80.0 ± 4.0 ^a^	75.0 ± 3.0 ^a^	62.0 ± 4.0 ^a^	54.0 ± 3.0 ^a^
Water	0.5	32.0 ± 2.5 ^d^	28.0 ± 2.0 ^d^	20.0 ± 2.0 ^d^	16.0 ± 2.0 ^d^
	1.0	52.0 ± 3.0 ^c^	46.0 ± 3.0 ^c^	36.0 ± 3.0 ^c^	30.0 ± 2.5 ^c^
	2.0	70.0 ± 4.0 ^b^	65.0 ± 3.0 ^b^	52.0 ± 3.0 ^b^	44.0 ± 3.0 ^b^
	4.0	86.0 ± 3.0 ^a^	80.0 ± 3.0 ^a^	68.0 ± 4.0 ^a^	58.0 ± 3.0 ^a^

Values expressed as mean ± SD, of three independent replicates. Different superscript letters (^a^ > ^b^ > ^c^ > ^d^) within each column indicate statistically significant differences between concentrations (p < 0.05, Tukey’s HSD).

The progressive increase in mycelial inhibition along the solvent polarity gradient—hexane < chloroform < ethanol < water—mirrors the pattern previously established for the antibacterial activity of these extracts and strongly suggests that the principal antifungal constituents of *C. ladanifer* are concentrated in its polar fractions. At the highest concentration tested (4.0 mg/mL), inhibition of *T. rubrum* increased from 48.0% ± 3.0% for the hexane extract to 68.0% ± 4.0%, 80.0% ± 4.0%, and 86.0% ± 3.0% for the chloroform, ethanolic, and aqueous extracts, respectively, representing an overall 1.8-fold enhancement in potency across the polarity range. This gradient is consistent with the preferential enrichment of polar antimicrobial compound classes—notably hydrolyzable tannins, ellagic acid derivatives, gallic acid, and flavonoid glycosides—in the ethanolic and aqueous fractions of *Cistus* species, all of which have been shown to interfere with fungal cell membrane integrity, inhibit ergosterol biosynthesis, and suppress the activity of cell wall-synthesizing enzymes including chitin synthase and β-1,3-glucan synthase (Lin-Vien et al., 1991). The particularly strong activity of the aqueous extract, recording inhibition values of 86.0% ± 3.0% and 80.0% ± 3.0% against *T. rubrum* and *Microsporum canis* at 4.0 mg/mL, may be related to the selective solubilization in hot water of high-molecular-weight ellagitannins and galloylglucoses that have been reported to exert membrane-disrupting effects on dermatophytic fungi through their capacity to complex with ergosterol and to denature cell surface glycoproteins essential for hyphal elongation and spore germination (Andrade et al., 2009; Amensour et al., 2010).

Notwithstanding the dominance of polar extracts, the hexane fraction exhibited antifungal activity that, while modest in absolute terms, was nonetheless consistently measurable across all four fungal species and all concentrations tested—a finding that contrasts markedly with its near-absent antibacterial activity against Gram-negative organisms reported earlier in this study. This divergence is mechanistically justified by the fundamental difference in target site between antibacterial and antifungal modes of action: bacterial outer membranes, composed predominantly of phospholipids and lipopolysaccharides, are poor targets for the terpenoids and diterpenes dominant in non-polar *Cistus* fractions, whereas fungal cytoplasmic membranes are rich in ergosterol—a sterol absent in prokaryotes—which constitutes a high-affinity binding target for lipophilic terpenoid compounds. Labdanolic acid and related labdane-type diterpenes, which represent characteristic constituents of the hexane fraction of *C. ladanifer* (Barrajón-Catalán et al., 2011; Abdelfattah et al., 2024), are well established to intercalate into ergosterol-containing membranes, inducing permeability dysregulation and ultimately fungal cell lysis at concentrations achievable *in vitro*. The chloroform fraction, extracting compounds of intermediate polarity including flavonoid aglycones, coumarins, and certain sesquiterpenes, displayed intermediate antifungal activity (4.0 mg/mL: 68.0% ± 4.0% against *T. rubrum*), consistent with the contribution of both lipophilic terpenoid and moderately polar phenolic constituents to the overall antifungal profile of this extract.

The susceptibility pattern observed across fungal species—*T. rubrum* > *M. canis* > *B. cinerea* > *F. oxysporum*—was maintained with remarkable consistency across all four extracts and all four concentration levels, and reflects well-established differences in cell wall architecture, membrane composition, and intrinsic resistance mechanisms among these organisms. Dermatophytic fungi, including *T. rubrum* and *M. canis*, are keratinophilic pathogens whose cell walls, while composed of chitin and glucan polymers, are structurally less complex and less densely cross-linked than those of phytopathogenic molds, rendering them more accessible to the penetration and membrane-disrupting activity of plant-derived bioactive compounds. *Trichophyton rubrum* in particular, as the most clinically prevalent dermatophyte responsible for the majority of onychomycosis and tinea pedis cases globally, is characteristically susceptible to polyphenolic compounds that target its ergosterol-dependent membrane permeability and inhibit the keratinolytic enzyme systems responsible for its pathogenicity. The slightly lower inhibition values recorded for *M. canis* relative to *T. rubrum* across all extracts may reflect differences in conidial wall thickness, melanin content, or efflux pump expression between the two dermatophyte species, though both remained significantly more susceptible than the phytopathogens across the full experimental range. *Botrytis cinerea* and *F. oxysporum*, as soil-borne necrotrophic plant pathogens, possess heavily melanized and chitinized hyphal walls that confer substantially greater intrinsic resistance to exogenous antimicrobial compounds, with *F. oxysporum* consistently recording the lowest inhibition values across all conditions—a finding concordant with the documented tolerance of this organism to a broad spectrum of natural and synthetic antifungal agents attributable to its constitutively active detoxification enzymes and thickened cell wall structure.

The strict monotonicity of the concentration-inhibition relationship across all extract-strain combinations, confirmed by statistically significant differences between all four concentration levels (p < 0.05, Tukey’s HSD), provides robust evidence of genuine dose-dependent pharmacological activity rather than non-specific cytotoxic effects, and is consistent with a receptor-mediated or membrane-targeted mode of action in which increasing concentrations of bioactive compounds progressively saturate available binding sites on the fungal cell surface. The standard deviations recorded across all conditions (±1.0% to ±4.0%) reflect acceptable biological variability for *in vitro* mycelial growth inhibition assays conducted in triplicate, and the absence of any anomalous or non-monotonic data points across the 64 individual measurements strengthens confidence in the reproducibility and reliability of the experimental results. Taken together, these findings establish *C. ladanifer* Soxhlet extracts—and in particular the aqueous and ethanolic fractions—as promising sources of broad-spectrum antifungal agents active against both clinically relevant dermatophytes and economically important phytopathogenic fungi, and provide a compelling rationale for the bioactivity-guided isolation of the specific polar constituents responsible for the observed activity, their structural characterization, and the determination of minimum fungicidal concentrations (MFC) and EC_50_ values to enable rigorous pharmacological benchmarking against established antifungal standards such as fluconazole and amphotericin B.

Consistent with the percentage-inhibition data, the interpolated EC_50_ values reproduced both the polarity ranking (water < ethanol < chloroform < hexane) and the species-susceptibility order (T. rubrum > *M. canis* > *B. cinerea* > F. oxysporum). For the aqueous fraction, EC_50_ values were 0.93, 1.16, 1.83, and 2.69 mg/mL against T. rubrum, *M. canis*, *B. cinerea*, and F. oxysporum, respectively, and for the ethanolic fraction 1.08, 1.36, 2.21, and 3.28 mg/mL; the hexane fraction did not reach 50% inhibition against any species within the tested range (EC_50_ > 4 mg/mL), as was also the case for the chloroform fraction against *B. cinerea* and F. oxysporum. The complete EC_50_ matrix is provided in Supplementary Table S5. Minimum fungicidal concentrations (MFC) were not determined in the present mycelial-growth assay and are identified as a priority endpoint for future work.

### 4.4 ADMET analysis

ADMET evaluation is a critical component of the present computational workflow because docking affinity alone cannot define the biological relevance of a phytochemical candidate. A compound with a favorable docking score may still be limited by poor oral absorption, excessive polarity, low aqueous solubility, unfavorable permeability, metabolic liability, transporter-mediated efflux, or predicted toxicity. Therefore, the ADMET layer was used to contextualize the selected Cistus ladanifer metabolites before interpreting their target-binding potential. This is particularly important for natural products, which often combine strong bioactivity with complex physicochemical properties that can strongly influence exposure, distribution, and developability (Bitew et al., 2021; Alotaibi et al., 2024; Hammouti et al., 2025).

**FIGURE 6:**
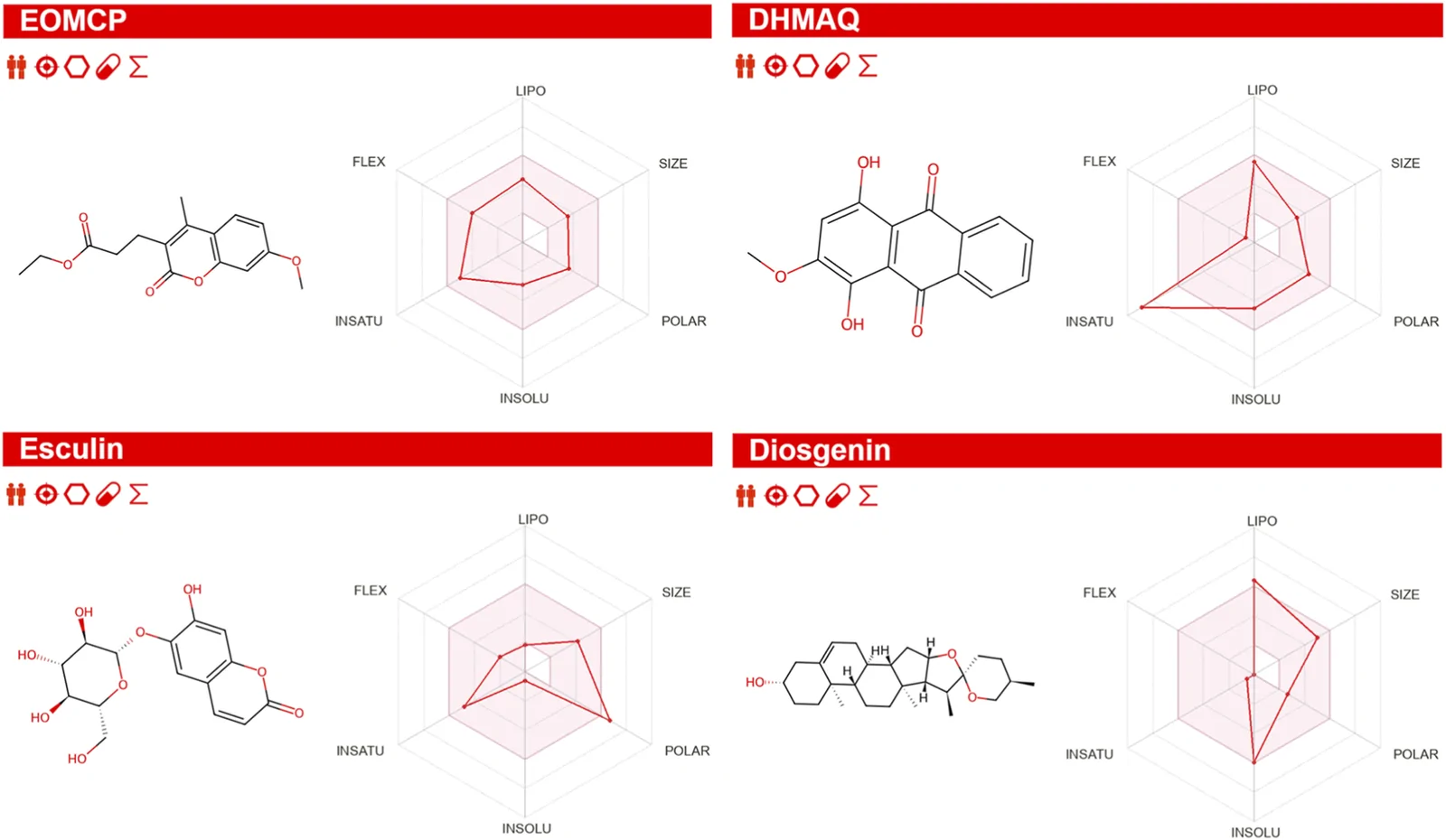
SwissADME bioavailability radar profiles of EOMCP, DHMAQ, Esculin, and Diosgenin.

Figure 6 provides a rapid physicochemical visualization of the drug-likeness space occupied by the four selected molecules. EOMCP shows the most balanced radar profile, with lipophilicity, polarity, size, solubility, saturation, and flexibility remaining close to the recommended region. This balanced profile is consistent with its moderate molecular weight, acceptable TPSA, absence of hydrogen-bond donors, and intermediate consensus LogP, suggesting that EOMCP has a structurally favorable profile for passive oral absorption. DHMAQ also remains largely compatible with the acceptable drug-like space, although its anthraquinone scaffold and higher polarity slightly shift the molecule toward a more rigid and polar profile. Esculin displays the most pronounced deviation in the polarity axis because of its glycosidic structure, high number of hydrogen-bonding groups, and elevated TPSA, which explains its weaker predicted oral absorption. Diosgenin shows the opposite behavior: its steroidal scaffold produces high lipophilicity and low polarity, supporting membrane permeability but also indicating poor aqueous solubility and a potential developability limitation.

Table 4 confirms that EOMCP and DHMAQ have the most conventional oral drug-likeness profiles among the aromatic compounds, with molecular weights below 300 g/mol, no Lipinski or Veber violations, moderate TPSA values, and high predicted gastrointestinal absorption. EOMCP is particularly balanced from a SwissADME perspective, whereas DHMAQ combines high intestinal absorption with a near-neutral BBB logBB value among the available pkCSM outputs, although its lower solubility may require attention in future formulation or analog optimization (Tlemcani et al., 2025; Benabbou et al., 2026). Esculin remains fully compliant with Lipinski criteria, but its high TPSA of 149.82 Å^2^, five hydrogen-bond donors, nine acceptors, negative consensus LogP, Veber violation, and reduced predicted intestinal absorption indicate that its glycosylated character favors aqueous compatibility at the expense of passive membrane permeation. Diosgenin shows high predicted gastrointestinal absorption and low TPSA, but this favorable permeability profile is counterbalanced by high lipophilicity, very low water solubility, one Lipinski violation, predicted CYP3A4 substrate behavior, and renal OCT2 substrate liability. Overall, Table 4 separates the compounds into three pharmacokinetic patterns: balanced oral candidates represented by EOMCP and DHMAQ, a highly polar and poorly permeable glycoside represented by Esculin, and a lipophilic membrane-permeable but poorly soluble steroidal scaffold represented by Diosgenin.

**TABLE 4:** In silico ADME, drug-likeness, permeability, metabolism, and clearance descriptors for selected Cistus ladanifer compounds predicted by SwissADME and pkCSM.

Parameter	EOMCP	DHMAQ	Esculin	Diosgenin
Section 1: Physicochemical properties				
Molecular weight (g/mol)	290.31	270.24	340.28	414.62
H-bond acceptors	5	5	9	3
H-bond donors	0	2	5	1
Rotatable bonds	6	1	3	0
Molar refractivity	79.42	70.29	78.65	121.59
TPSA (Å^2^)	65.74	83.83	149.82	38.69
Consensus log P (o/w)	2.32	2.24	−1.80	5.15
Log S (w)	−2.92	−4.51	−0.61	−5.98
Section 2: Drug-likeness and oral bioavailability				
GI absorption	High	High	Low	High
Lipinski violations	0	0	0	1
Veber violations	0	0	1	0
Bioavailability score	0.55	0.55	0.55	0.55
Intestinal absorption (human, %)	100	95.991	50.784	97.028
Section 3: BBB and CNS penetration				
BBB permeability (log BB)	−0.542	−0.002	−1.191	0.291
CNS permeability (log PS)	−2.869	−2.310	−4.027	−2.936
Section 4: Metabolic and transporter interactions				
CYP2D6 inhibitor	No	No	No	No
CYP3A4 inhibitor	No	No	No	No
CYP2D6 substrate	No	No	No	No
CYP3A4 substrate	No	No	No	Yes
Section 5: Excretion and clearance				
Renal OCT2 substrate	No	No	No	Yes
Total clearance (log ml/min/kg)	0.968	0.076	0.681	0.328

**FIGURE 7:**
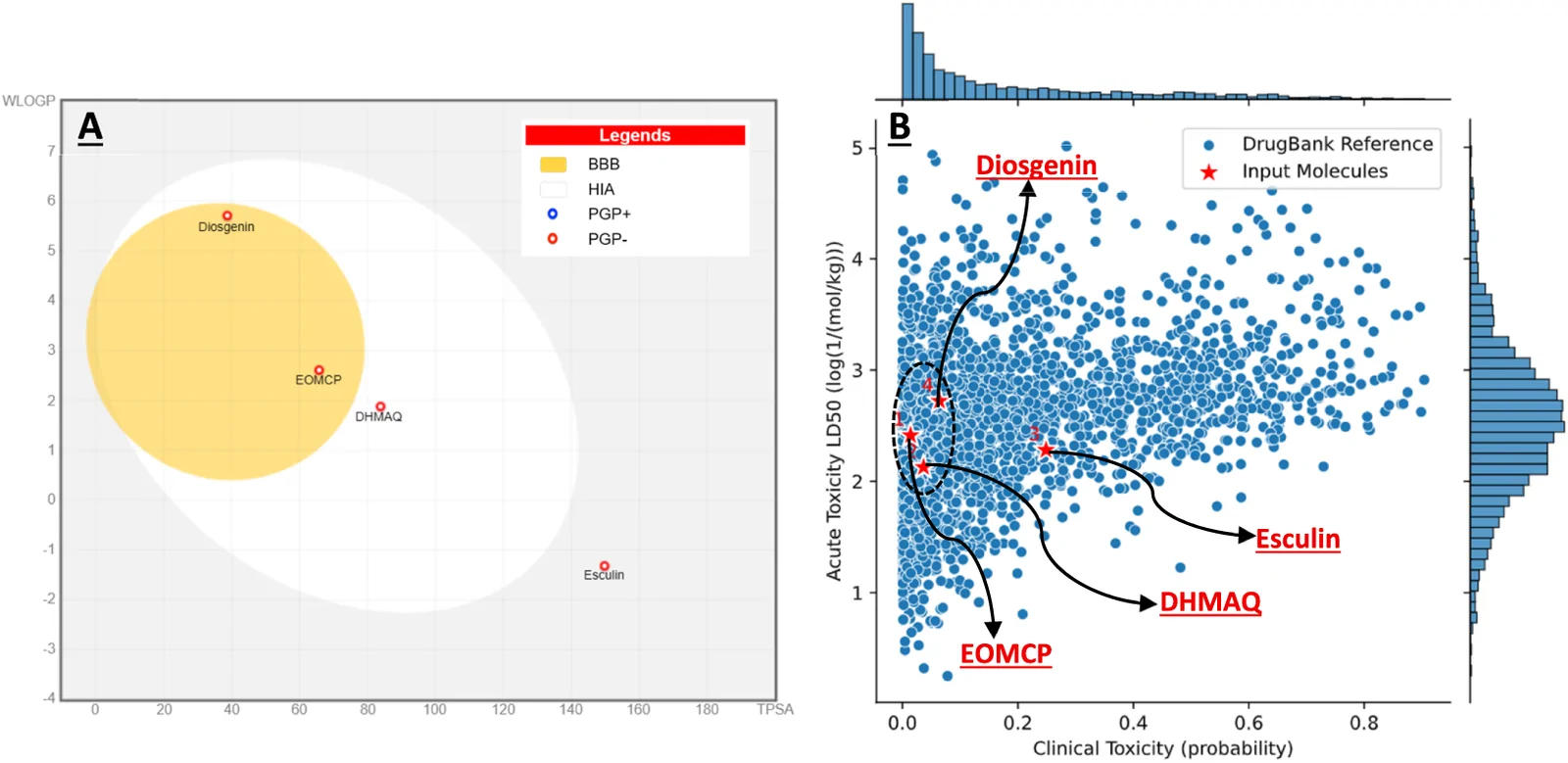
Integrated ADMET visualization of the selected compounds: **(A)** SwissADME BOILED-Egg model for gastrointestinal absorption, blood-brain barrier permeation, and P-gp status; **(B)** ADMET-AI projection of acute toxicity and clinical toxicity probability relative to DrugBank reference molecules.

Figure 7A further illustrates the permeability consequences of these physicochemical differences. Both EOMCP and Diosgenin are positioned in the yellow BBB-permeant region of the BOILED-Egg plot, consistent with their favorable balance between polarity and lipophilicity for passive membrane diffusion. EOMCP and DHMAQ are also located within the favorable human intestinal absorption region, supporting their predicted high gastrointestinal absorption and confirming that both compounds occupy an acceptable oral-absorption space. However, DHMAQ remains outside the central yellow BBB region, suggesting a more limited CNS distribution than EOMCP and Diosgenin. Esculin is displaced toward the high-TPSA region, outside the optimal absorption space, confirming that its extensive hydroxylation and glycosidic architecture restrict passive permeability. Figure 7B complements this absorption analysis by mapping the same molecules in the ADMET-AI toxicity space. The compounds remain embedded within the main DrugBank reference distribution rather than appearing as isolated outliers, but they occupy distinct risk zones. EOMCP lies close to the low clinical-toxicity probability region, DHMAQ and Esculin occupy intermediate positions, and Diosgenin shows a higher acute-toxicity coordinate in this projection (Malik et al., 2017; Dexlin et al., 2021). These toxicity predictions should therefore be interpreted as prioritization signals requiring experimental verification, but they are useful for ranking compounds before deeper biological testing.

### 4.5 Molecular docking

Molecular docking was used to complement the experimental antimicrobial and antifungal findings by evaluating whether the selected phytochemicals could fit into key bacterial and fungal protein pockets. The target panel included 1KZN, an *E. coli* DNA gyrase B domain involved in bacterial DNA supercoiling and replication, 3G7B, a *Staphylococcus aureus* DNA gyrase B ATP-binding target essential for bacterial DNA replication, 5FRB, *Aspergillus fumigatus* CYP51B/sterol 14α-demethylase involved in fungal ergosterol biosynthesis, and 8JZN, fungal 1,3-β-glucan synthase involved in fungal cell-wall β-glucan production (Ciocarlan et al., 2024; Sargsyan, 2025; Vibha et al., 2025). Thus, docking was used as a hypothesis-generating approach to support ligand–pocket complementarity and identify the most plausible compound–target pairs, without replacing biochemical inhibition assays (de Azevedo, 2019; Yahaya, 2020; Prabhakar et al., 2016).

Table 5 shows a coherent target-dependent docking pattern in which 5FRB emerges as the most favorable predicted binding environment for all four compounds. Diosgenin produced the strongest score of the full series against 5FRB (−12.7 kcal/mol), slightly exceeding the co-crystallized reference ligand (−12.5 kcal/mol), which suggests an excellent steric and hydrophobic match between the steroidal scaffold and this pocket. Esculin also displayed a strong 5FRB score (−9.3 kcal/mol), followed by DHMAQ (−8.8 kcal/mol) and EOMCP (−8.4 kcal/mol), indicating that both polar coumarin-like scaffolds and lipophilic steroidal architecture can be accommodated in this fungal target. For the bacterial targets, DHMAQ ranked highest against 1KZN (−8.0 kcal/mol), whereas Esculin produced the best score against 3G7B (−8.2 kcal/mol), slightly better than the corresponding co-crystallized reference value (−8.1 kcal/mol). Docking against 8JZN was generally weaker, although Diosgenin remained the best-scoring compound (−6.9 kcal/mol), exceeding the reference ligand value (−6.4 kcal/mol). These data collectively suggest that the antifungal-associated target 5FRB is the most permissive and energetically favorable pocket for the selected metabolites, while the bacterial pockets show more selective accommodation of aromatic and glycosylated scaffolds.

**TABLE 5:** Molecular docking binding affinities (kcal/mol) of selected Cistus ladanifer compounds and co-crystallized reference ligands against antibacterial and antifungal targets.

Selected ligand	Chemical class	1KZN	3G7B	5FRB	8JZN	Best predicted target
EOMCP	Chromene/coumarin derivative	−7.5	−7.6	−8.4	−5.5	5FRB
DHMAQ	Hydroxylated anthraquinone	−8.0	−8.1	−8.8	−6.0	5FRB
Esculin	Coumarin glycoside	−7.8	−8.2	−9.3	−5.8	5FRB
Diosgenin	Steroidal sapogenin	−7.9	−6.3	−12.7	−6.9	5FRB
Co-crystallized ligand	References ligand	−9.4	−8.1	−12.5	−6.4	5FRB

These docking and ADMET results are strictly hypothesis-generating and do not, in themselves, validate biological activity. Two caveats are important. First, the strongest experimental activity across the antioxidant and antimicrobial assays resides in the aqueous fraction, whereas the strongest docking score is obtained for diosgenin, a minor constituent (1.68%) detected only in the ethanol fraction and absent from the aqueous extract; docking therefore does not account for the activity of the most potent fraction, which is most plausibly driven by the non-GC-amenable polyphenols not represented in the docking panel. Second, the four docked compounds were selected for scaffold diversity and tractability, not because they are the quantitatively dominant constituents. Accordingly, the compounds examined here should be regarded as candidate scaffolds for hypothesis-driven follow-up rather than as validated lead compounds, and target-based enzyme-inhibition assays would be required before any lead claim could be substantiated.

**FIGURE 8:**
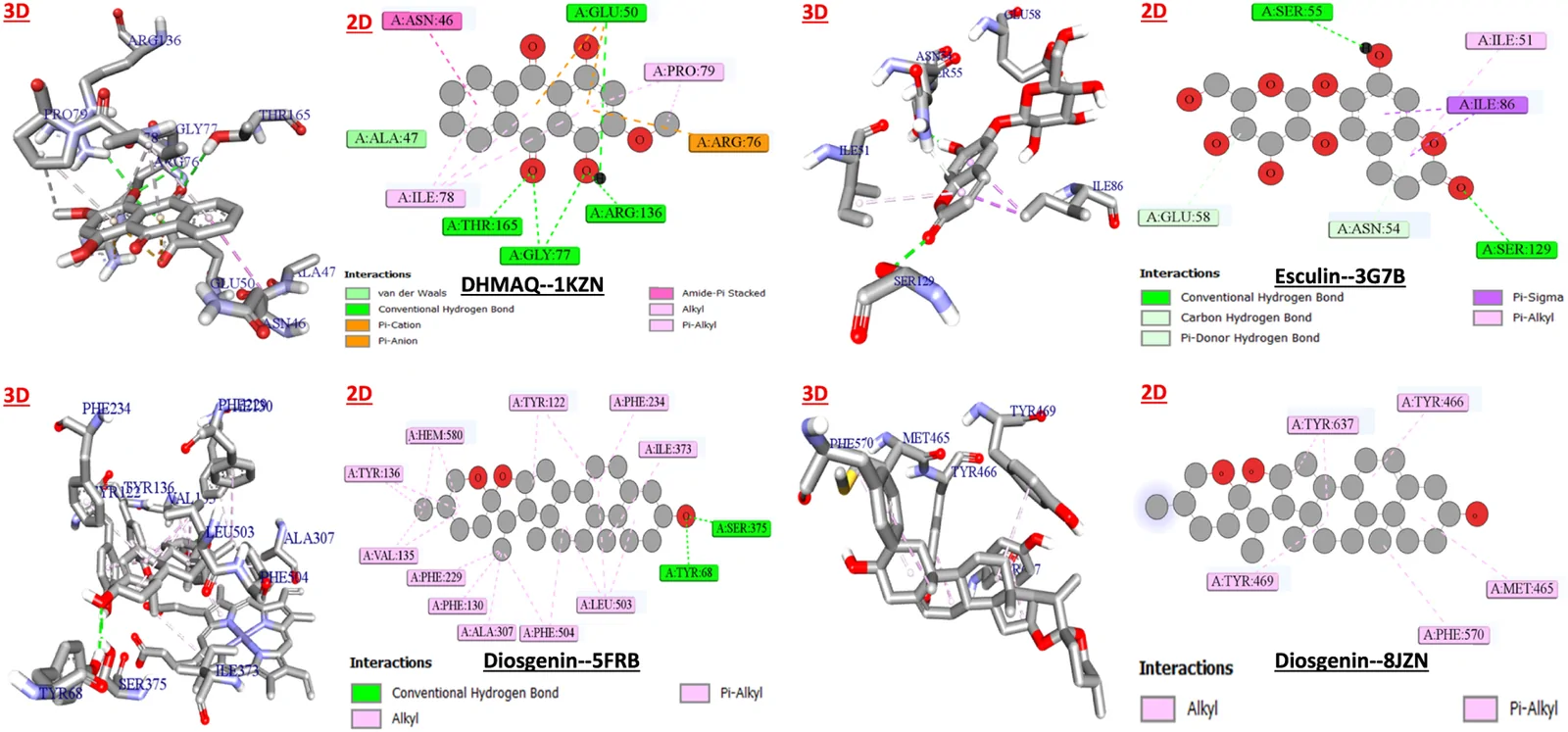
Three-dimensional binding poses and two-dimensional interaction maps for the best-scoring compound-target pairs: DHMAQ-1KZN, Esculin-3G7B, Diosgenin-5FRB, and Diosgenin-8JZN.

Figure 8 explains the structural basis of the main docking trends summarized in Table 5. In the DHMAQ-1KZN complex, the hydroxylated anthraquinone core is stabilized by a mixed polar and aromatic interaction network involving GLU50, ARG136, GLY77, THR165, ASN46, ARG76, ILE78, PRO79, and ALA47. The coexistence of hydrogen bonds, pi-cation/pi-anion, amide-pi, alkyl, and pi-alkyl contacts rationalizes the favorable DHMAQ score because the planar anthraquinone scaffold can function both as a polar anchoring platform and as an aromatic contact surface (Asiamah et al., 2023; Hussain et al., 2020; Tina et al., 2026). In the Esculin-3G7B complex, SER55, GLU58, ASN54, and SER129 provide a strong polar anchoring network, while ILE51 and ILE86 stabilize the coumarin nucleus through hydrophobic and pi-associated contacts. This explains why Esculin performs well against 3G7B despite its high polarity: the sugar moiety supports hydrogen bonding, whereas the coumarin ring contributes pocket recognition.

The Diosgenin-5FRB complex gives the clearest structural explanation for the strongest docking score. Its steroidal skeleton is deeply buried in a hydrophobic/aromatic environment involving TYR122, PHE234, ILE373, LEU503, PHE504, PHE130, PHE229, ALA307, VAL135, and the heme-associated region, while SER375 and TYR68 provide limited but meaningful polar orientation. In Diosgenin-8JZN, stabilization is mainly alkyl/pi-alkyl in character through TYR637, TYR466, TYR469, MET465, and PHE570, with fewer polar contacts; this explains the weaker 8JZN score. Overall, the 2D/3D interaction analysis supports the docking ranking by showing that the best scores arise from chemically coherent ligand-pocket complementarity.

### 4.6 Study limitations

Several limitations of the present study should be acknowledged and delineate priorities for future work. First, the bioactivity assays were performed on whole solvent fractions rather than on isolated compounds; consequently, the assignment of specific activities to individual constituents is inferential and supported by the phytochemical gradient and literature evidence rather than by bioactivity-guided isolation. Second, the antimicrobial screening relied on agar diffusion and broth microdilution endpoints; mechanistic interpretations regarding bacterial membrane disruption, *P. aeruginosa* efflux- and permeability-mediated resistance, and the antifungal modes of action invoked above (ergosterol binding and inhibition of chitin synthase and β-1,3-glucan synthase) are drawn from established microbiology and were not experimentally dissected here—confirmatory assays such as time–kill kinetics, propidium iodide uptake, ATP leakage, and efflux-inhibitor synergy testing are required to validate them. Third, the *in silico* ADMET and molecular docking analyses are predictive prioritization tools and do not substitute for enzyme-inhibition assays or experimentally determined binding constants. Finally, and importantly, no cytocompatibility or *in vivo* toxicity data were generated in this study; the ADMET-AI toxicity outputs are computational estimates only. Accordingly, the translational and applied claims made here should be regarded as preliminary, and cellular cytotoxicity, selectivity-index determination, and *in vivo* safety and efficacy evaluation are necessary prerequisites before any pharmaceutical, nutraceutical, or cosmetic application can be substantiated.

## 5 Conclusion

The present study provides the first comprehensive, polarity-resolved phytochemical and bioactivity profile of *Cistus ladanifer* L. leaves, achieved through sequential Soxhlet extraction and integrated phytochemical, spectroscopic, and biological characterization. The results collectively establish a robust and internally coherent polarity–activity framework that rationalizes the observed bioactivity in terms of the chemically defined fractions obtained. Several principal conclusions can be drawn from this work.

Sequential Soxhlet extraction achieved effective and reproducible chemical partitioning of the plant matrix into four pharmacologically meaningful domains, as confirmed by FTIR spectroscopy and GC-MS analysis. The hexane fraction concentrated an epicuticular wax alkane assemblage dominated by tetracosane (71.20%), with minor contributions from fatty acid derivatives and diterpenes. The chloroform fraction was distinguished by a chromene derivative (ethyl 3-(2-oxo-7-methoxy-4-methylchromen-3-yl)propionate, 10.74%), a hydroxylated anthraquinone (5,8-dihydroxy-6-methoxy-9,10-anthraquinone, 0.77%), and monoterpenes of documented antimicrobial relevance. The ethanolic fraction was dominated by the cyclitol myo-inositol (41.60%), D-mannonic acid (23.41%), and a diverse suite of shikimate intermediates, glycosylated phenylpropanoids (esculin), and the steroidal sapogenin diosgenin. The aqueous fraction concentrated D-glucuronic acid (73.18%) alongside organic acids, reflecting the plant’s primary metabolic and cell-wall carbohydrate pool. The GC–MS analysis provided a tentative phytochemical profile of the different solvent fractions and, together with the spectrophotometric determination of total phenolics, flavonoids, and tannins, helped contextualize the observed biological activities, although it does not identify the specific compounds responsible for these effects.

A consistent and statistically significant polarity-dependent bioactivity gradient was demonstrated across all three biological assay categories. Phytochemical quantification confirmed that phenolic compounds, flavonoids, and condensed tannins are predominantly hydrophilic, with the aqueous fraction accumulating the highest total phenolic (140 mg GAE/g DW), flavonoid (148 mg QE/g DW), and tannin (210 mg TAE/g DW) contents. Concordantly, the aqueous fraction exhibited the strongest antioxidant activity across all four mechanistic assays—including a DPPH IC50 of 0.09 ± 0.001 mg/mL and a FRAP value of 65.25 ± 0.2 mg TE/g DW—values comparable to or exceeding those reported for benchmark antioxidant-rich plant extracts such as pomegranate peel and green tea. The multi-mechanistic nature of this activity, spanning both electron transfer (DPPH, ABTS, FRAP) and hydrogen atom transfer (ORAC) pathways, is characteristic of complex polyphenolic mixtures and substantially strengthens the biological relevance of the polar fractions. Antibacterial activity followed the same gradient, with the aqueous extract recording the lowest MIC values overall (0.25 mg/mL against both *S. aureus* and *B. subtilis*), and all active fractions were classified as bactericidal based on MBC/MIC ratios of 2–4. Similarly, antifungal mycelial growth inhibition increased monotonically with solvent polarity, reaching up to 86.0% ± 3.0% against *T. rubrum* at 4.0 mg/mL for the aqueous fraction, with dermatophytes consistently more susceptible than phytopathogenic fungi across all tested fractions. These bioactivity gradients are mechanistically consistent with the established antimicrobial and antioxidant pharmacology of polar polyphenolic compound classes—notably hydrolyzable tannins, ellagic acid derivatives, and flavonoid glycosides—and are fully congruent with the ethnobotanical tradition of preparing *C. ladanifer* as a hot aqueous infusion or decoction in Moroccan and Mediterranean folk medicine.

The hexane fraction, while markedly inferior to the polar fractions in antibacterial potency, demonstrated consistently measurable antifungal and antioxidant activity attributable to labdane-type diterpenes and lipophilic terpene derivatives that target the ergosterol-containing fungal membrane—a mechanism distinct from that operating in the polar fractions and suggesting complementary rather than redundant biological roles across the chemical domains of this plant. The non-polar fraction may therefore be of particular relevance in lipophilic biological compartments such as cell membranes, where polar polyphenolics are less effective, and warrants independent investigation for topical antifungal applications.

In silico ADMET analysis identified EOMCP and DHMAQ as the most balanced oral drug candidates among the selected marker compounds, with high predicted gastrointestinal absorption, no Lipinski violations, and favorable clearance profiles. Esculin, while compliant with Lipinski’s rule of five, is limited by high polarity and reduced membrane permeability attributable to its glycosidic architecture, whereas diosgenin’s lipophilic steroidal scaffold, while conferring high membrane permeability, imposes solubility and CYP3A4-related metabolic liabilities that would require formulation optimization. Molecular docking established that the antifungal target CYP51B (5FRB) constitutes the most accommodating binding environment for all four compounds, with diosgenin producing the strongest binding affinity across the entire series (−12.7 kcal/mol), exceeding the co-crystallized reference ligand and supporting a plausible ergosterol biosynthesis inhibition mechanism. Taken together, the *in silico* data provide a translational bridge between the experimental bioactivity findings and target-level molecular interactions, while highlighting the need for enzymatic inhibition assays and cellular mechanistic studies to validate these computational predictions.

Overall, the experimental and computational evidence presented in this study firmly establishes the polar Soxhlet fractions of Moroccan *C. ladanifer* as promising sources of broad-spectrum bioactive phytochemicals with antioxidant, bactericidal, and antifungal activities that, being derived from *in vitro* data, warrant cytocompatibility and *in vivo* safety evaluation before application. The convergence of experimental data, mechanistic reasoning, and *in silico* profiling provides a rigorous and reproducible foundation for the rational valorization of this ethnomedicinally important species in pharmaceutical, nutraceutical, and cosmetic applications. Future investigations should prioritize bioactivity-guided isolation and structural elucidation of the principal polar bioactive constituents, determination of structure–activity relationships across the identified polyphenolic series, evaluation of antioxidant efficacy and antimicrobial mechanisms in relevant cellular models, and assessment of *in vivo* efficacy and safety to bridge the gap between the *in vitro* findings reported here and therapeutic applicability.

## Data Availability

The original contributions presented in the study are included in the article/Supplementary Material, further inquiries can be directed to the corresponding author.
