# Supplementary material for: Sequential soxhlet fractionation of Cistus ladanifer L. reveals a polarity–activity gradient: GC-MS profiling, multimechanistic bioactivity, and in silico identification of antifungal and antibacterial lead compounds
Source: Front Chem. 2026 Aug 5;14:1909751. doi: 10.3389/fchem.2026.1909751 (PMC13487886; doi:10.3389/fchem.2026.1909751)
Supplement: Supplementary file 1 [file DataSheet1.docx]

**Supplementary Material**

[**Sequential Soxhlet Fractionation of Cistus ladanifer L. Reveals a Polarity–Activity Gradient: GC-MS Profiling, Multimechanistic Bioactivity, and In Silico Identification of Antifungal and Antibacterial Lead Compounds**](https://review.frontiersin.org/Document/DownloadPDF?articleId=1909751&userId=3486460&roleId=16)

**1. GC-MS total ion chromatograms**

**Supplementary Figure S1.** GC-MS total ion chromatogram of the hexane (Hex) Soxhlet fraction of *Cistus ladanifer* leaves

**Supplementary Figure S2.** GC-MS total ion chromatogram of the chloroform (Chl) Soxhlet fraction of *Cistus ladanifer* leaves

**Supplementary Figure S3.** GC-MS total ion chromatogram of the ethanol (EtOH) Soxhlet fraction of *Cistus ladanifer* leaves, with the corresponding solvent blank.

**Supplementary Figure S4.** GC-MS total ion chromatogram of the aqueous (AQ) Soxhlet fraction of *Cistus ladanifer* leaves

**2. Antifungal EC₅₀ values**

**Supplementary Table S5.** Half-maximal effective concentrations (EC₅₀, mg/mL) of the four *Cistus ladanifer* leaf Soxhlet fractions against dermatophytic and phytopathogenic fungi.

| Fraction | *T. rubrum* | *M. canis* | *B. cinerea* | *F. oxysporum* |
| --- | --- | --- | --- | --- |
| Hexane | >4 | >4 | >4 | >4 |
| Chloroform | 1.84 | 2.38 | >4 | >4 |
| Ethanol | 1.08 | 1.36 | 2.21 | 3.28 |
| Water | 0.93 | 1.16 | 1.83 | 2.69 |

*EC₅₀ (mg/mL) were interpolated from the mycelial-inhibition dose–response data (0.5–4.0 mg/mL) on a log₂-concentration scale. “>4” indicates that 50% inhibition was not reached within the tested concentration range. Values are derived from assays performed in triplicate.*
